# Patterns of Z chromosome divergence among *Heliconius* species highlight the importance of historical demography

**DOI:** 10.1111/mec.14560

**Published:** 2018-04-14

**Authors:** Steven M. Van Belleghem, Margarita Baquero, Riccardo Papa, Camilo Salazar, W. Owen McMillan, Brian A. Counterman, Chris D. Jiggins, Simon H. Martin

**Affiliations:** ^1^ Department of Zoology University of Cambridge Cambridge UK; ^2^ Department of Biological Sciences Mississippi State University Mississippi State MS USA; ^3^ Department of Biology Center for Applied Tropical Ecology and Conservation University of Puerto Rico Rio Piedras Puerto Rico; ^4^ Smithsonian Tropical Research Institute Apartado Panamá Panama; ^5^ Biology Program Faculty of Natural Sciences and Mathematics Universidad del Rosario Carrera, Bogota Colombia

**Keywords:** absolute divergence measures, demography, *Heliconius*, large‐X effect, relative divergence measures, speciation

## Abstract

Sex chromosomes are disproportionately involved in reproductive isolation and adaptation. In support of such a “large‐X” effect, genome scans between recently diverged populations and species pairs often identify distinct patterns of divergence on the sex chromosome compared to autosomes. When measures of divergence between populations are higher on the sex chromosome compared to autosomes, such patterns could be interpreted as evidence for faster divergence on the sex chromosome, that is “faster‐X”, barriers to gene flow on the sex chromosome. However, demographic changes can strongly skew divergence estimates and are not always taken into consideration. We used 224 whole‐genome sequences representing 36 populations from two *Heliconius* butterfly clades (*H. erato* and *H. melpomene*) to explore patterns of Z chromosome divergence. We show that increased divergence compared to equilibrium expectations can in many cases be explained by demographic change. Among *Heliconius erato* populations, for instance, population size increase in the ancestral population can explain increased absolute divergence measures on the Z chromosome compared to the autosomes, as a result of increased ancestral Z chromosome genetic diversity. Nonetheless, we do identify increased divergence on the Z chromosome relative to the autosomes in parapatric or sympatric species comparisons that imply postzygotic reproductive barriers. Using simulations, we show that this is consistent with reduced gene flow on the Z chromosome, perhaps due to greater accumulation of incompatibilities. Our work demonstrates the importance of taking demography into account to interpret patterns of divergence on the Z chromosome, but nonetheless provides evidence to support the Z chromosome as a strong barrier to gene flow in incipient *Heliconius* butterfly species.

## 1 INTRODUCTION

Comparisons between genomes of diverging populations or species have revealed elevated differentiation on the sex chromosomes in several animals, such as flycatchers (Ellegren et al., 2012), crows (Poelstra et al., 2014), Darwin's finches (Lamichhaney et al., 2015), ducks (Lavretsky et al., 2015) and *Heliconius* butterflies (Kronforst et al., 2013; Martin et al., 2013; Van Belleghem et al., 2017). These patterns of elevated sex chromosome divergence are sometimes readily interpreted as the result of increased reproductive isolation and reduced admixture on the sex chromosomes and, thus, ascribed to a large‐X effect (Box 1). However, it remains unresolved whether such elevated sex‐linked divergence actually results from more rapid accumulation of isolating barriers on the sex chromosome or could be explained by differences in effective population size between the sex chromosomes and the autosomes (Meisel & Connallon, 2013; Pool & Nielsen, 2007; Wolf & Ellegren, 2017).

Box 1 **Consequences of Hemizygous Sex Chromosomes**

**Large‐x or z Effect and What can Cause It**

Sex chromosomes have been repeatedly shown to have a disproportionate role during speciation (Coyne & Orr, 2004), demonstrated by three widespread intrinsic postmating effects (Johnson & Lachance, 2012; Turelli & Moyle, 2007): (i) Haldane's rule, (ii) reciprocal‐cross asymmetry of hybrid viability and sterility and (iii) the large‐X effect. *Haldane's rule* states that where only one sex of the hybrids has reduced viability or fertility, that sex is most commonly the heterogametic sex (Haldane, 1922). *Asymmetry of hybrid viability and sterility* refers to the situation where reciprocal crosses often differ in their incompatibility phenotype (Turelli & Moyle, 2007). Finally, the *large‐X effect* highlights the disproportionate contribution of the sex chromosomes to the heterogametic and asymmetric inviability/sterility in hybrids (Coyne & Orr, 1989).

Haldane's rule can generally be explained by between‐locus “Bateson‐Dobzhansky–Muller incompatibilities” (BDMs) (Dobzhansky, 1935; Muller, 1942; Orr, 1996), in which divergent alleles at different loci become fixed between populations and cause inappropriate epistatic interactions only when brought together in novel hybrid allele combinations (Coyne & Orr, 2004). If interacting loci include recessive alleles on the sex chromosome, only the heterogametic sex will suffer incompatibilities in the F1 generation (Turelli & Orr, 1995). Additionally, BDMs between autosomal loci and the sex chromosomes can be specific to a particular direction of hybridization due to their uniparental inheritance and, thus, also explain asymmetric reproductive isolation (Turelli & Moyle, 2007). Hence, hemizygous expression of recessive alleles on the sex chromosome has been put forward as a cause for the disproportionate role of the sex chromosomes during speciation (dominance theory) (Turelli & Orr, 1995).

In contrast to the dominance theory, there is, however, a large body of observations and theory that propose alternative or additional explanations that can cause a large‐X effect and/or explain Haldane's rule (Presgraves, 2008; Wu & Davis, 1993). These factors include faster male evolution resulting from intense sexual selection among males (Wu & Davis, 1993), meiotic drive (Frank, 1991), dosage compensation (Jablonka & Lamb, 1991) and faster‐X evolution (Charlesworth, Coyne, & Barton, 1987; Vicoso & Charlesworth, 2006). Faster male evolution targets genes with male‐biased effects, which may be sex‐linked or autosomal, in which case it can cause Haldane's rule only when males are the heterogametic sex. By contrast, faster‐X evolution does not necessarily depend on selection related to sex and is predicted if adaptive new mutations are on average partially recessive and thus are presented more readily to selection on the hemizygous sex chromosomes (Charlesworth et al., 1987). In *Drosophila*, faster‐X evolution has been studied extensively. Although it is not ubiquitous, there is clear evidence for faster‐X divergence and adaptation (Counterman, Ortíz‐Barrientos, & Noor, 2004), particularly for X‐linked genes expressed in male reproductive tissues (reviewed in Meisel & Connallon, 2013). In Lepidoptera (butterflies and moths), Haldane's rule and the large‐X (or Z) effect have been reported for numerous species (Presgraves, 2002; Prowell, 1998; Sperling, 1994). As lepidopteran females are heterogametic ZW, while males are ZZ, the Z is equivalent to the X. However, as females are heterogametic, faster male evolution is insufficient to explain Haldane's, but faster‐X evolution remains a viable explanation (Sackton et al., 2014). Moreover, in Lepidoptera, the large‐X effect extends beyond intrinsic isolating barriers and there are differences in many traits and behaviours that map disproportionately to the Z chromosome (Prowell, 1998; Sperling, 1994). These observations are consistent with the faster accumulation of differences on the Z chromosome (faster‐X evolution).

**Factors Affecting Sex/Autosome Diversity Ratios**

Apart from population size changes, factors that can result in deviations from the expected three‐quarter X/autosome (X/A) diversity ratio, and could thus potentially affect divergence measures, include (i) sex‐biased demographic events leading to different effective population sizes of males and females (Charlesworth, 2001), (ii) selective sweeps and background selection differently affecting the sex chromosomes (Charlesworth, 2012) and (iii) differences in mutation rates between sexes or between the sex chromosomes and the autosomes (Johnson & Lachance, 2012; Sayres & Makova, 2011).

First, different population sizes of males and females can influence the X/A diversity ratio because two‐thirds of the X chromosome population is transmitted through females. A male‐biased population would thus decrease the X/A diversity ratio below three‐quarters, whereas a female‐biased population would increase the ratio. This effect would be opposite in female heterogametic sex systems (ZW).

Second, the hemizygous expression of the sex chromosome could result in both higher purifying selection and more efficient selection of beneficial recessive mutations (~selective sweeps) and result in a decrease in the expected X/A diversity ratio (Charlesworth et al., 1987). Additionally, differences in recombination rates can lead to different extent of loss of variation through linked selection and thus background selection (Charlesworth, 2012). In Lepidoptera, meiosis is commonly achiasmatic (no recombination) in the heterogametic sex (females) (Suomalainen, Cook, & Turner, 1973; Turner & Sheppard, 1975). A reduction in recombination rate on the sex chromosomes compared to autosomes, which is commonly found in *Drosophila* (Vicoso & Charlesworth, 2009), should thus not be expected to decrease Z/A diversity ratios through increased background selection in *Heliconius*. On the other hand, it has been suggested that effective recombination should be higher, and thus background selection lower, for the Z chromosome when recombination is absent in females (Charlesworth, 2012). This is because the Z chromosomes spend two‐thirds of their time in recombining males, whereas autosomes only spend half of their time in recombining males.

Third, because the male germ line generally involves more cell divisions and thus opportunities for replication errors, sex‐linked genes may have different mutation rates. Because X‐linked genes spend only one‐third of their time in males and two‐thirds of their time in females, the X chromosome may be subjected to a lower mutation rate. Conversely, the Z chromosome spends two‐thirds of its time in males and may therefore become enriched in genetic variation compared to the autosomes (Johnson & Lachance, 2012; Sayres & Makova, 2011; Vicoso & Charlesworth, 2006). Such increased mutation rates on the Z chromosome could also increase the rate of divergence between Z chromosomes (Kirkpatrick & Hall, 2004).

FIGURE B1 The effect of population size on the coalescent and measures of diversity and divergence. The branches represent two populations, 1 and 2, that have split at a certain time (grey dashed line). This branching event occurs on two chromosomes that have a different population size, such as the autosomes (grey) and X chromosome (green). The black lines show the coalescent of two alleles in each population. The branches show that the coalescence process is influenced by the split time as well as the population size. Population size affects the nucleotide diversity within each population (π), the total nucleotide diversity (π_T_) and absolute divergence *d*_XY_, but not *d*_a_ as indicated by the vertical coloured lines. For *d*_a_, the average of the within‐population nucleotide diversity (π_S_) is used as the estimate of the ancestral nucleotide diversity (π_anc_). The influence of population size on *F*_ST_ can be seen as resulting from a decrease in the denominator (π_T_), but not in the numerator (π_T_ and π_s_ change proportionately)

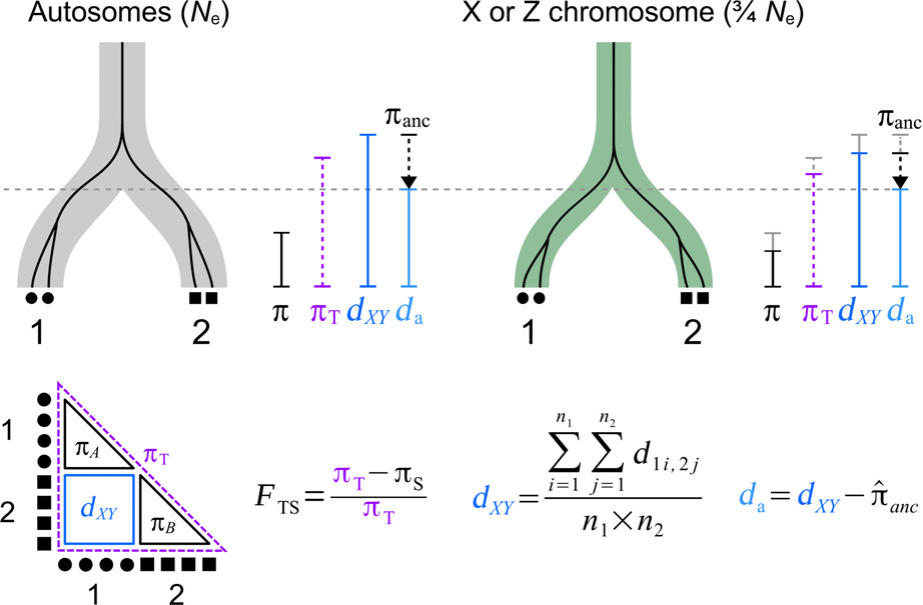

When comparing divergence between genomic regions, such as sex chromosomes versus autosomes, measures of population divergence are influenced by within‐population diversity (Charlesworth, 1998; Cruickshank & Hahn, 2014) (Box 2). This is explicitly the case for relative measures such as *F*_ST_, but also less directly for absolute measures of divergence such as *d*_XY_. For absolute divergence measures, this is because the genetic divergence between two alleles sampled from two species includes both divergence accumulated postspeciation, but also diversity already present in the ancestral population before the split. The latter is strongly dependent on effective population size. In a population under equilibrium conditions where the two sexes have an identical distribution of offspring number, the X chromosome‐effective population size and genetic diversity is expected to be three‐quarters that of the autosomes. Deviations from this ratio can result from multiple unique features of the sex chromosomes (Box 1), and population size changes in particular can have strong differential influence on sex chromosome compared to autosomal diversity (Pool & Nielsen, 2007). Previous studies attempted to control for differences in effective population size on the sex chromosome, for instance among recently diverged duck species from Mexico, but such studies generally do not account for population size changes (Lavretsky et al., 2015). In order to interpret both relative and absolute measures of divergence on the sex chromosomes as evidence of a disproportionate contribution to species divergence and/or reduced admixture, we need to also account for demographic changes that can influence diversity of the sex chromosomes.

Box 2 **Measures of Divergence Depend on Population Size**

1 The mutational diversity in present‐day samples is directly related to population size, structure and age. This is because population size determines the rate of coalescence within and between populations (Figure B1). This relationship can be seen with *F*_ST_, which was developed to measure the normalized difference in allele frequencies between populations (Wright, 1931). The dependence of *F*_ST_ on population size can be understood by interpreting *F*_ST_ as the rate of coalescence within populations compared to the overall coalescence rate (Slatkin & Voelm, 1991). Comparing pairs of populations with different effective population sizes will therefore show distinct *F*_ST_ estimates even when the split time is the same (Charlesworth, 1998). Absolute divergence *d*_XY_ is the average number of pairwise differences between sequences sampled from two populations (Nei & Li, 1979). The measure *d*_XY_ is not influenced by changes to within‐population diversity that occur after the split, but does depend on diversity that was present at the time the populations split (Gillespie & Langley, 1979). Therefore, population pairs that had a smaller population size at the time they split will, consequently, have smaller *d*_XY_ estimates. To compare pairs of populations that had different ancestral population sizes, *d*_a_ has been proposed, which subtracts an estimate of the diversity in the ancestral population from the absolute divergence measure *d*_XY_ (Nei & Li, 1979). An approximation of ancestral diversity can be obtained by taking the average of the nucleotide diversity observed in the two present‐day populations. Such a correction should result in the “net” nucleotide differences that have accumulated since the time of split.

To show how these different divergence measures perform, we simulated a simplified population split with varying degrees of migration (*m*) (Figure B2). As expected, the values *F*_ST_, *d*_XY_ and *d*_a_ all increase when migration between populations decreases. *F*_ST_ and *d*_XY_ are clearly influenced by population size. While for *d*_XY_ *,* this results from the variation present at the time of the split, *F*_ST_ does not show a simple linear relationship with population size. Only *d*_a_ represents the net accumulation of differences that can be compared between populations of different sizes, such as the sex chromosomes versus autosomes (but see section 3.3 in Results and discussion).

FIGURE B2 Simulated effect of population size differences on divergence measures *F*_ST_, *d*_XY_ and *d*_a_. Simulations were performed for two populations that split 4 million generations ago and vary in their degree of migration (*m*). A lower effective population size, such as for the X chromosome (green) compared to autosomes (grey), results in higher *F*_ST_ and lower *d*_XY_ estimates, but has no effect on *d*_a_ under these assumptions

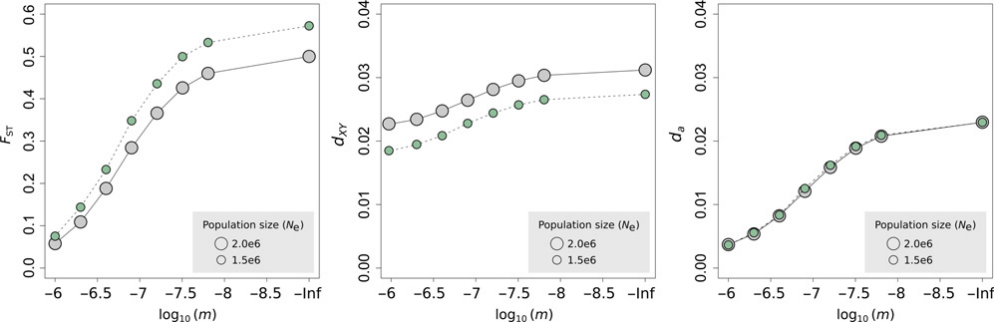

**Figure 1:**
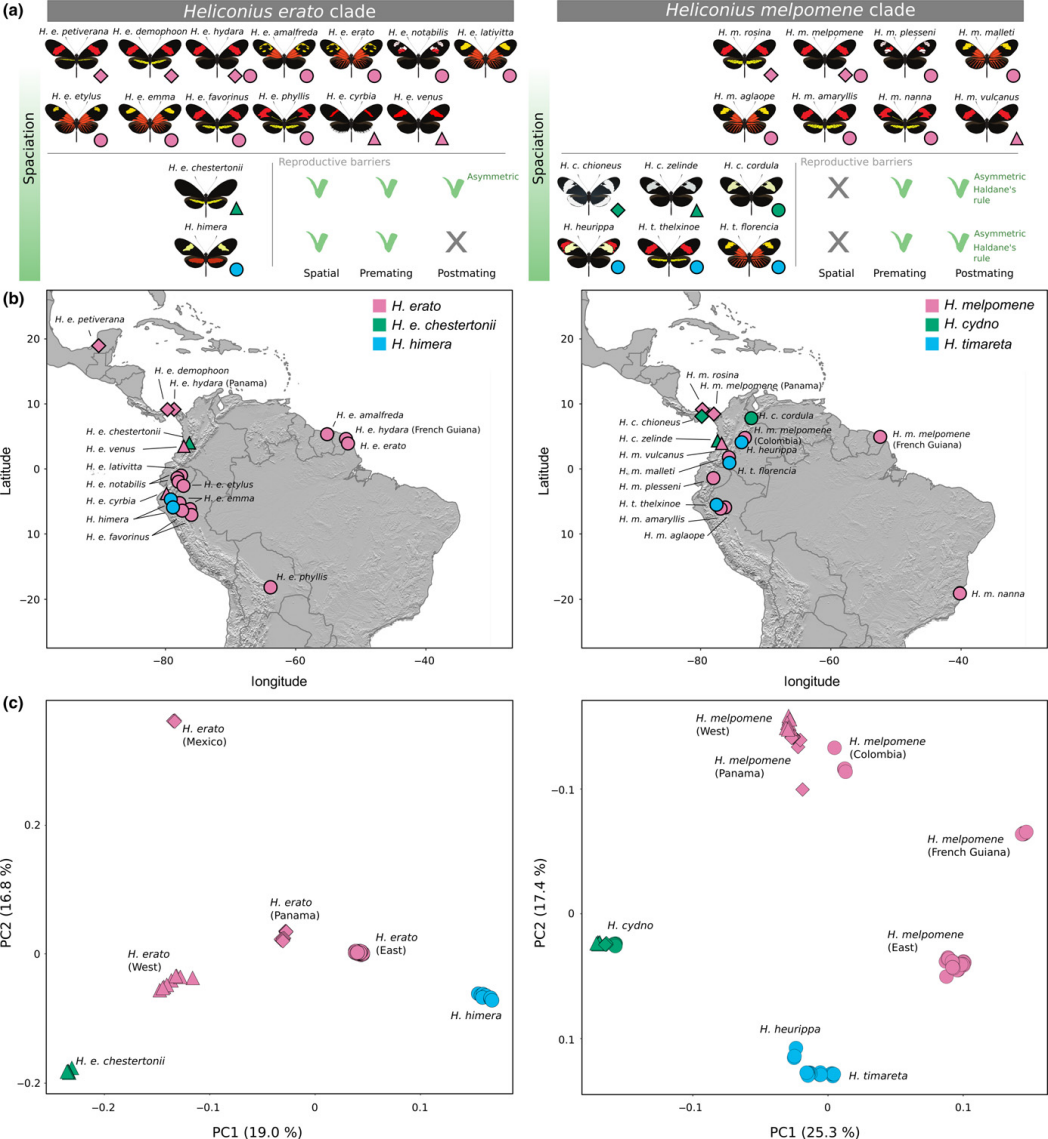
Diversity and sampling of the *Heliconius erato* and *Heliconius melpomene* clade. (a) *Heliconius erato chestertonii* (green) is reproductively isolated from *H. erato* (pink) by spatial separation (parapatry), mate choice and (asymmetric) reduced hybrid fertility of both sexes (i.e., no Haldane's rule). *Heliconius himera* (blue) is reproductively isolated from *H. erato* by spatial separation and mate choice, but hybrids show no reduced fertility. *Heliconius cydno* (green) and *H. timareta* (blue) occur sympatrically with *H. melpomene* (pink) populations, but are both reproductively isolated by strong mate choice and (asymmetric) reduced fertility of F1 hybrids (i.e., Haldane's rule). (b) Localities of sampled populations included in this study. Within *H. erato*,* H. melpomene*,* H. timareta* and *H. cydno* names represent different races that display distinct colour patterns. Shapes represent geographic regions: Mexico and Panama (diamond), west of the Andes (triangles) and east of the Andes (circles). (c) PCA plots of autosomal SNP variation. Note that *H. m. nanna* has not been included in the PCA as the signal of geographic isolation between *H. m. nanna* and the other populations dominates the signal (see Fig. S1) [Colour figure can be viewed at http://wileyonlinelibrary.com]

Here, we explore diversity and divergence on the Z chromosome relative to the autosomes among populations of the *Heliconius erato* and *Heliconius melpomene* butterfly clades, using these different measures. The *H. erato* and *H. melpomene* clades diverged about 13 million years ago (Kozak et al., 2015) and represent unpalatable and warningly coloured butterflies that have independently radiated into many divergent geographic races and reproductively isolated species. Within both clades, speciation has been accompanied by shifts in Müllerian mimicry (Mallet, McMillan, & Jiggins, 1998), and where populations come into contact, hybrid phenotypes usually have reduced survival rates due to strong frequency‐dependent selection against intermediate colour pattern phenotypes (Jiggins, Mcmillan, Neukirchen, Mallet, & Nw, 1996; Mallet & Barton, 1989; Merrill et al., 2012; Naisbit, Jiggins, & Mallet, 2001). Two species, *H. himera* and *H. e. chestertonii*, are geographic replacements of *H. erato* in dry Andean valleys. They are partially reproductively isolated, but individuals of hybrid ancestry make up about 10% of the population in narrow transition zones between forms (McMillan, Jiggins, & Mallet, 1997; Merrill, Chia, & Nadeau, 2014; Muñoz, Salazar, Castaño, Jiggins, & Linares, 2010). Similarly, *H. cydno* and *H. timareta* are geographic replacements of each other and both are broadly sympatric with *H. melpomene*. Here, both species are reproductively isolated from *H. melpomene* by a combination of pre‐ and postmating isolation (Mérot, Salazar, Merrill, Jiggins, & Joron, 2017). Species integrity does not seem to involve structural variation such as chromosomal inversions (Davey et al., 2017). Instead, reproductive barriers include strong selection against hybrids, mate choice and postzygotic incompatibilities (Figure 1a). Assortative mating has evolved in both the *H. erato* and *H. melpomene* clades (Jiggins, Naisbit, Coe, & Mallet, 2001; McMillan et al., 1997; Merrill et al., 2014; Muñoz et al., 2010). In the *H. erato* clade, sterility and reciprocal‐cross asymmetry of hybrid sterility have been reported in crosses between *H. erato* and *H. e. chestertonii* (Muñoz et al., 2010), but hybrid sterility is absent between *H. erato* and *H. himera* (McMillan et al., 1997). In the *H. melpomene* clade, female sterility (Haldane's rule; Box 1) and reciprocal‐cross asymmetry of hybrid sterility occur in crosses between *H. melpomene* and *H. cydno* (Naisbit, Jiggins, Linares, Salazar, & Mallet, 2002), *H. melpomene* and *H. heurippa* (Salazar et al., 2005) and *H. melpomene* and *H. timareta* (Sánchez et al., 2015), as well as between allopatric *H. melpomene* populations from French Guiana and those from Panama and Colombia (Jiggins, Linares et al., 2001). In support of a large‐X effect, sterility in these crosses (*H. melpomene*×* H. cydno, H. melpomene* ×* H. heurippa* and *H. melpomene* ×* H.timareta*) was found to be Z‐linked.

The presence of incipient species pairs with different levels of reproductive isolation allows us to examine the relative rate of autosomal and Z chromosomal evolution and the factors that are likely influencing patterns of divergence. We take advantage of a large genomic data set composed of 224 whole genomes representing 20 populations of the *H. erato* clade and 16 populations of the *H. melpomene* clade. We also use simulations to evaluate the effect that demographic changes have on the estimate of relative rates of divergence on the Z versus the autosomes and demonstrate that in many comparisons, demography can explain much of the observed elevated divergence on the Z relative to the autosomes. However, by taking into account geographic distance or autosomal divergence as a proxy for gene flow, we show that there is evidence for increased divergence on the Z chromosome for species pairs with known postzygotic reproductive barriers. These rates of increased divergence likely reflect reduced admixture on the Z chromosome and provide support for the Z chromosome being a greater barrier to gene flow in some incipient *Heliconius* butterfly species.

## 2 MATERIALS AND METHODS

### 2.1 Sampling

We used whole‐genome resequenced data of a total of 109 butterflies belonging to the *Heliconius erato* clade and 115 from the *Heliconius melpomene* clade (Figure 1; Tables S1 and S2). The *H. erato* clade samples comprised fifteen colour pattern forms from twenty localities: *Heliconius erato petiverana* (Mexico, *n* = 5), *Heliconius erato demophoon* (Panama, *n* = 10), *Heliconius erato hydara* (Panama, *n* = 6 and French Guiana, *n* = 5), *Heliconius erato erato* (French Guiana, *n* = 6), *Heliconius erato amalfreda* (Suriname, *n* = 5), *Heliconius erato notabilis* (Ecuador *Heliconius erato lativitta* contact zone, *n* = 5 and Ecuador *Heliconius erato etylus* contact zone, *n* = 5), *H. e. etylus* (Ecuador, *n* = 5), *H. e. lativitta* (Ecuador, *n* = 5), *Heliconius erato emma* (Peru *H. himera* contact zone, *n* = 4 and Peru *Heliconius erato favorinus* contact zone, *n* = 7), *H. e. favorinus* (Peru *H. himera* contact zone, *n* = 4 and Peru *H. e. emma* contact zone, *n* = 8), *Heliconius erato phyllis* (Bolivia, *n* = 4), *Heliconius erato venus* (Colombia, *n* = 5), *Heliconius erato cyrbia* (Ecuador, *n* = 4), *Heliconius erato chestertonii* (Colombia, *n* = 7) and *H. himera* (Ecuador *H. e. emma* contact zone*, n* = 5, and Peru *H. e. cyrbia* contact zone, *n* = 4).

The *H. melpomene* clade samples comprised fourteen colour pattern forms from sixteen localities (Figure 1; Tables S2). Ten populations were sampled from the *H. melpomene* clade: *H. m. melpomene* (Panama, *n* = 3), *H. m. melpomene* (French Guiana, *n* = 10), *H. m. melpomene* (Colombia, *n* = 5), *H. m. rosina* (Panama, *n* = 10), *H. m. malleti* (Colombia, *n* = 10), *H. m. vulcanus* (Colombia, *n* = 10), *H. m. plesseni* (Ecuador, *n* = 3), *H. m. aglaope* (Peru, *n* = 4), *H. m. amaryllis* (Peru, *n* = 10), and *H. m. nanna* (Brazil, *n* = 4). Three populations were sampled from the *H. timareta* clade: *H. heurippa* (Colombia, *n* = 3), *H. t. thelxinoe* (Peru, *n* = 10) and *H. t. florencia* (Colombia, *n* = 10). Three populations were sampled from the *H. cydno* clade: *H. c. chioneus* (Panama, *n* = 10), *H. c. cordula* (Venezuela, *n* = 3) and *H. c. zelinde* (Colombia, *n* = 10).

### 2.2 Sequencing and genotyping

Whole‐genome 100‐bp paired‐end Illumina resequencing data from *H. erato* and *H. melpomene* clade samples were aligned to the *H. erato* v1 (Van Belleghem et al., 2017) and *H. melpomene* v2 (Davey et al., 2016) reference genomes, respectively, using bwa v0.7 (Li, 2013). PCR duplicated reads were removed using picard v1.138 (http://picard.sourceforge.net) and sorted using samtools (Li et al., 2009). Genotypes were called using the genome analysis tool kit (GATK) Haplotypecaller (Van der Auwera et al., 2013). Individual genomic VCF records (gVCF) were jointly genotyped using GATK's genotype GVCFs. Genotype calls were only considered in downstream analysis if they had a minimum depth (DP) ≥ 10, maximum depth (DP) ≤ 100 (to avoid false SNPs due to mapping in repetitive regions), and for variant calls, a minimum genotype quality (GQ) ≥ 30. The W chromosome has not been identified in *Heliconius*, but read depth comparisons between Z and autosomes in males and females (e.g., see supplementary material Martin et al., 2013) support the hypothesis that there is no significant mapping of W‐linked reads to the Z and the W is, thus, unlikely to interfere with genotyping. The absence of mapping of W‐linked reads to the Z is likely due to the degenerate sequence and highly repetitive nature of the W chromosome. The data set contained 31 and 11 female samples (ZW) randomly distributed among the *H. erato* and *H. melpomene* clade populations, respectively (Tables S1 and S2). These samples had lower read and, consequentially, lower genotyping coverage for the Z chromosome, but using the stringent filter thresholds, this does not affect variant and nonvariant sites differently and does not affect measures of nucleotide diversity and divergence.

### 2.3 Population structure and historical demography

To discern population structure among the sampled *H. erato* and *H. melpomene* clade individuals, we performed principal component analysis (PCA) using EIGENSTRAT SmartPCA (Price et al., 2006). For this analysis, we only considered autosomal biallelic sites that had coverage in all individuals.

We inferred changes in the historical population size from individual consensus genome sequences using pairwise sequentially Markovian coalescent (PSMC) analyses as implemented in MSMC (Schiffels & Durbin, 2014). This method fits a model of changing population size by estimating the distribution of times to the most recent common ancestor along diploid genomes. When used on single diploid genomes, this method is similar to pairwise sequentially Markovian coalescent (PSMC) analyses (Li & Durbin, 2011). Genotypes were inferred from bwa v0.7 (Li, 2013) mapped reads separately from previous genotyping analysis using samtools v0.1.19 (Li et al., 2009) according to authors' suggestions. This involved a minimum mapping (‐q) and base (‐Q) quality of 20 and adjustment of mapping quality (‐C) 50. A mask file was generated for regions of the genome with a minimum coverage depth of 30 and was provided together with heterozygosity calls to the MSMC tool. MSMC was run on heterozygosity calls from all contiguous scaffolds longer than 500 kb, excluding scaffolds on the Z chromosome. We scaled the PSMC estimates using a generation time of 0.25 years and a mutation rate of 2e‐9 as estimated for *H. melpomene* (Keightley et al., 2014).

### 2.4 Population genomic diversity and divergence statistics

We first estimated diversity within populations as well as divergence between parapatric and sympatric populations in nonoverlapping 50‐kb windows along the autosomes and Z chromosome using python scripts and egglib (data presented in Figure 3, 7 and S4–6) (De Mita & Siol, 2012). We only considered windows for which at least 10% of the positions were genotyped for at least 75% of the individuals within each population. For females, haploid was enforced when calculating divergence and diversity statistics. Sex of individuals was inferred from heterozygosity on the Z. *F*_ST_ was estimated as in Hudson, Slatkin, and Maddison (1992), as

$$F_{\mathrm{ST}}=\frac{\pi_{T}-\pi_{s}}{\pi_{T}},$$

with nucleotide diversity in a population (π_*i*_) calculated as

$$\pi_{i}=\frac{\sum_{j=1}^{n_{i}-1}\sum_{k=j+1}^{n_{i}}d_{ij,lk}}{\left(\begin{array}{c} n_{i}\\ 2 \end{array}\right)}$$

and average within‐population nucleotide diversity (π_*S*_) calculated as the weighted (*w*) average of the nucleotide diversity (π_i_) within each population *l* and *k*, as

$$\pi_{S}=w\pi_{1}+\left(1-w\right)\pi_{2}.$$

Total nucleotide diversity (π_*T*_) was calculated as the average number of nucleotide differences per site between two DNA sequences in all possible pairs in the sampled population (Hudson et al., 1992), as

$$\pi_{T}=\frac{\sum_{i=1}^{i=2}\sum_{j=1}^{n_{i}-1}\sum_{k=j+1}^{n_{i}}d_{ij,lk}+\sum_{i=1}^{n_{1}}\sum_{j=1}^{n_{2}}d_{1i,2j}}{\left(\begin{array}{c} n_{1}+n_{2}\\ 2 \end{array}\right)}.$$

Between‐population sequence divergence *d*_XY_ was estimated as the average pairwise difference between sequences sampled from two different populations (Nei & Li, 1979), as

$$d_{\mathrm{XY}}=\frac{\sum_{i=1}^{n_{1}}\sum_{j=1}^{n_{2}}d_{1i,2j}}{n_{1}+n_{2}}.$$

The relative measure of divergence *d*_*a*_ was calculated by subtracting *d*_XY_ with an estimate of the nucleotide diversity (π_*S*_) in the ancestral populations (Nei & Li, 1979),

$$d_{a}=d_{\mathrm{XY}}-\pi_{s}.$$

Tajima's *D* was calculated as a measure of deviation from a population evolving neutrally with a constant size, with negative values indicating an excess of rare alleles (~selective sweep or population expansion) and positive values indicating a lack of rare alleles (~balancing selection or population contraction) (Tajima, 1989). To overcome the problem of nonindependence between loci, estimates of the variance in nucleotide diversity (π) and Tajima's *D* within populations along the genomes were obtained using block‐jackknife deletion over 1‐Mb intervals along the genome (chosen to be much longer than linkage disequilibrium in *Heliconius* (Martin et al., 2013)) (Künsch, 1989).

To calculate pairwise *d*_XY_ values between each individual, we subsampled the genomes by only considering genomic sites that were at least 500 bp apart and had coverage for at least one individual in each population (data presented in Figures 8 and 9). For the *H. erato* clade data set, this resulted in a high coverage data set with 322,082 and 15,382 sites on the autosomes and Z chromosome, respectively. For the *H. melpomene* clade data set, this resulted in 335,636 and 18,623 sites on the autosomes and Z chromosome, respectively. Pairwise *d*_XY_ values between each individual were used to evaluate the relationship between absolute genetic divergence (*d*_XY_) and geographic distance using Mantel's tests (Mantel, 1967). Mantel's tests are commonly used to test for correlations between pairwise distance matrices and were performed using the R package vegan (Oksanen et al., 2016). Pairwise distances between populations were calculated from the average of the sample coordinates obtained for each population (Table S3, S4).

### 2.5 Simulations

To compare patterns in our data to expectations, we simulated genealogies in 50‐kb sequence windows under certain evolutionary scenarios. The simulations were performed with a population recombination rate (4*N*_e_ *r*) of 0.01 using the coalescent simulator *msms* (Ewing & Hermisson, 2010). Subsequently, from the simulated genealogies, we simulated 50‐kb sequences with a mutation rate of 2e‐9 a Hasegawa–Kishino–Yano substitution model using *seq‐gen* (Rambaut & Grass, 1997).

In a first set of simulations, we considered one population that underwent a single population size change of a magnitude (*x*) ranging from 0.01 to 100 and at a certain moment backwards in time (*t*). In a second set of simulations, we considered pairs of populations that were connected through migration (*m*) ranging from 0 to 1e‐6 and for which migration was reduced with a factor *d* on the Z chromosome. To compare changes in the nucleotide diversity on autosomes and the Z chromosome, we simulated the Z chromosome as a separate population for which the effective population size was set to three‐quarters that of the autosomal population.

To compare populations with a different effective population size (*N*_e_), such as the autosomes and the Z chromosome, we expressed time in generations and migration rates as a proportion of the effective population size. Comparable to the *Heliconius* sampling, we sampled five individuals from each population and ran 300 replicates for each parameter combination. Pseudocode to run the *msms* command lines are provided in Tables S5. Tajima's *D*, nucleotide diversity (π) and *d*_XY_ were calculated from the simulated sequences using python scripts and egglib (De Mita & Siol, 2012).

## 3 RESULTS AND DISCUSSION

### 3.1 Population structure and historical demography in *Heliconius erato* and *Heliconius melpomene*

We mapped a total of 109 *Heliconius erato* clade resequenced genomes to the *Heliconius erato* v1 reference genome (Van Belleghem et al., 2017) and 115 *Heliconius melpomene* clade genomes to the *Heliconius melpomene* v2 reference genome (Davey et al., 2016). These samples represent 20 *H. erato* clade and 16 *H. melpomene* clade populations covering nearly the entire geographic distribution of these species groups (Figure 1a and b).

Phylogenies from whole‐genome data for the *H. erato* clade and *H. melpomene* clade have been presented previously in Van Belleghem et al. (2017) and Martin et al. (2016), respectively. Given the difficulty in presenting phylogenies for hybridizing populations and species, we here instead summarized relationships using principal component analysis (PCA) of the autosomal SNP variation using EIGENSTRAT SmartPCA (Price et al., 2006). PCA grouped the *H. erato* clade samples mainly according to geography, apart from *H. himera* individuals from Ecuador and northern Peru and *H. e. chestertonii* from Colombia (Figure 1c). Four main geographic groups were apparent: populations from Mexico, Panama, west of the Andes and east of the Andes. *Heliconius erato* populations east of the Andes as far as 3000 km apart were closely clustered in the PCA. The separate grouping of *H. himera* and *H. e. chestertonii* individuals supports these populations as representing incipient species that maintain their integrity, despite ample opportunity for hybridization and gene flow (Arias et al., 2008; Jiggins et al., 1996; McMillan et al., 1997). In the PCA, *H. himera* was more closely related to the *H. erato* populations east of the Andes, whereas *H. e. chestertonii* was more closely related to the West Andean populations.

PCA of the *H. melpomene* clade grouped individuals from west of the Andes and Panama closely together, with *H. melpomene* from Colombia being most similar to this population pair (Figure 1c). *Heliconius melpomene* populations from east of the Andes further clustered in three distinct groups, largely in agreement with geographic distance: populations from the eastern slopes of the Andes, the French Guiana population and *H. m. nanna* from Brazil (Figures 1c and S1). While phylogenetic reconstructions have suggested that *H. melpomene* and the *Heliconius cydno/timareta* clades are reciprocally monophyletic (Dasmahapatra et al., 2012; Martin et al., 2013, 2016; Nadeau et al., 2013), such patterns are hard to interpret from the PCA and patterns of relatedness may be influenced by more recent admixture. Nevertheless, *H. cydno* and *Heliconius timareta* clustered distinctly. *Heliconius cydno* formed a distinct cluster with little difference between samples from Panama, west or east of the Andes. *Heliconius timareta* grouped most closely with *Heliconius heurippa*, consistent with previous analysis (Arias et al., 2014; Nadeau et al., 2013).

**Figure 2:**
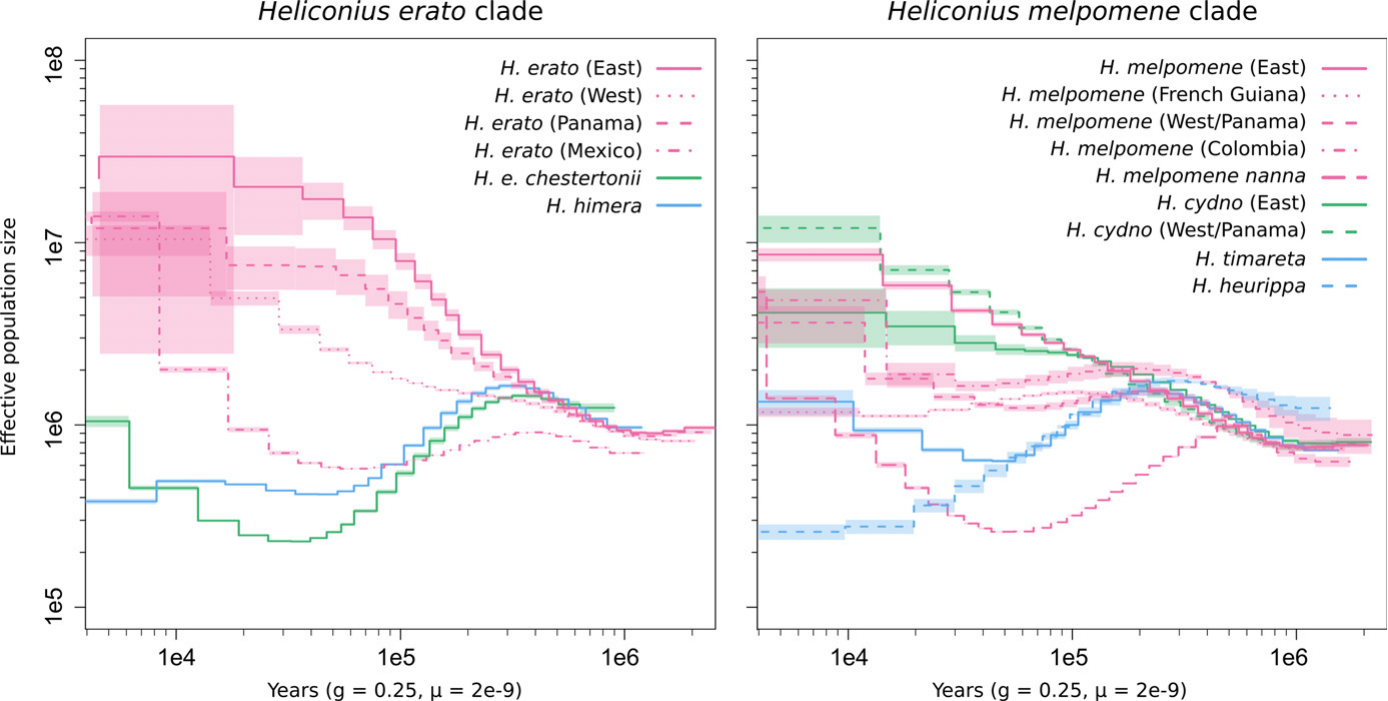
Inference of historical effective population size changes using pairwise sequentially Markovian coalescent (PSMC) analysis. Lines and shading represent the average effective population size and 95% CI, respectively, estimated from PSMC analyses on individual samples. The PSMC estimates are scaled using a generation time of 0.25 years and a mutation rate of 2e‐9. For PSMC results on each individual sample, see Figs S2 and S3 [Colour figure can be viewed at http://wileyonlinelibrary.com]

We inferred changes in the historical population size from individual consensus genome sequences using pairwise sequentially Markovian coalescent (PSMC) (Schiffels & Durbin, 2014) (Figures 2 and S2–3). *Heliconius erato* populations east of the Andes were inferred to have the strongest increase in population size, starting about 1 MYA. Similarly, but to a lesser extent, *H. erato* populations from west of the Andes and Panama are inferred to have had a continuous population size increase during the past million years. In contrast, after a period of population size increase, *H. himera* has undergone continuous population size decrease since about 300 KYA. The *H. e. chestertonii* population seems to have decreased in size since about 300 KYA and increased in size after 30 KYA. Note that all the population size estimates for *H. himera* and *H. e. chestertonii,* as well as *H. erato* from Panama and east and west of the Andes start to deviate about 300 to 400 KYA. In Mexico, the sampled *H. erato* population has undergone a more recent steep population size increase after a period of population decrease. The absence of convergence of the population size of the Mexican population with the other *H. erato* populations agrees with an old divergence of this population that likely falls out of the detection limit of the PSMC method (Van Belleghem et al., 2017).

In the *H. melpomene* clade, we find that *H. cydno* populations and *H. melpomene* from east of the Andes have undergone a continuous population size increase since about 1 MYA (Figure 2). In contrast, *H. melpomene* from French Guiana only shows this population size increase up to 100 KYA, after which it has experienced slight population size decrease. *Heliconius melpomene* populations from west of the Andes, Panama and Colombia show a more complex demographic history with population increase up to 300 KYA, followed by decrease and increase again about 30 to 40 KYA. *Heliconius melpomene nanna* from East Brazil is characterized by a steep population size decrease since 500 KYA and a steep population size increase starting 40–50 KYA. Next, *H. timareta* (i.e., *H. t. florencia* and *H. t. thelxinoe*) is characterized by population size increase until 200 to 300 KYA, followed by population size decrease and increase again about 40 KYA. Finally, *H. heurippa* has a similar demographic history as the other *H. timareta* species, but with a continuous decrease in population size since about 300 KYA.

The PSMC estimates give inference up to about 1 MYA (Figure 2). In contrast, the split time between *H. melpomene* and *H. cydno* has been estimated between 0.9 and 1.4 MYA ago (~3.6–5.6 million *Heliconius* generations) (Kronforst et al., 2013; Lohse, Chmelik, Martin, & Barton, 2016). A similar split time can be expected between *H. melpomene* and *H. timareta*, as *H. timareta* and *H. cydno* likely diverged after the split from *H. melpomene* (Martin et al., 2016). While such divergence time estimates that account for migration are unavailable for *H. e. chestertonii* and *H. himera*, their split times from *H. erato* are likely also older than most of the time interval in which historical demography is inferred by PSMC (Flanagan et al., 2004). Therefore, the PSMC results likely reflect only demographic changes that have occurred after these populations split. We also note that PSMC does not account for hybridization, which might impact the inferred histories. However, almost all sympatric and parapatric species pairs showed very different population histories to one another (e.g., *H. himera* and *H. e. cyrbia*;* H. t. thelxinoe* and *H. m. amaryllis*), suggesting that the differences observed between populations are largely driven by real demographic change rather than artefacts of hybridization.

### 3.2 Z chromosome divergence in *Heliconius erato* and *Heliconius melpomene*

**Figure 3:**
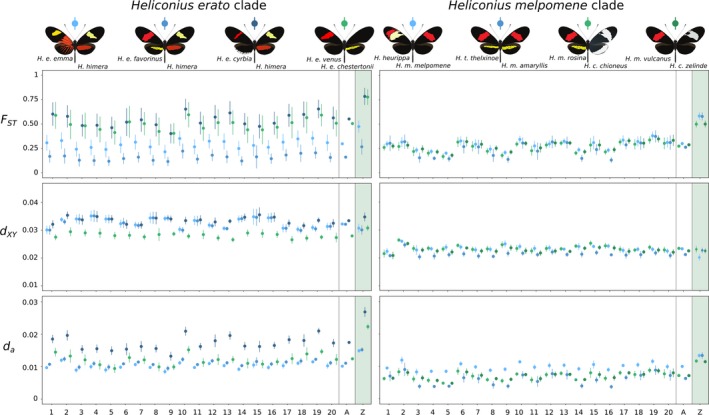
Averages of the divergence measures *F*_ST_,*d*_XY_ and *d*_a_ at the autosomes (1–20) and Z chromosome in four parapatric/sympatric species comparisons of the *Heliconius erato* and *Heliconius melpomene* clade. Averages for each chromosome and 95% confidence intervals (vertical bars) were estimated using block‐jackknifing with 1‐Mb intervals. The vertical dashed lines highlight the averages over all autosomes (a) and the estimates for the Z chromosome. Values were calculated in 50‐kb nonoverlapping windows along the chromosomes. For species relatedness, see PCA in Figure 1. Note that *H. e. emma* and *H. e. favorinus* represent closely related populations east of the Andes and are thus not completely independent comparisons to *Heliconius himera*. See Figs S4–S6 for stepping window plots of *F*_ST_,*d*_XY_ and *d*_a_ values, respectively [Colour figure can be viewed at http://wileyonlinelibrary.com]

To compare rates of divergence between the Z chromosome and autosomes, we calculated three commonly used measures of divergence, *F*_ST_, *d*_XY_ and *d*_a_ *,* between incipient species and population pairs of *H. erato* and *H. melpomene* (Figures 3 and S4–6). All three measures of sequence divergence are calculated from mutational diversity in the data, but are each dependent on population size in different ways (Box 2). In *Heliconius*,* F*_ST_ has been frequently used to identify regions in the genome under strong divergent selection (Martin et al., 2013; Nadeau et al., 2012; Van Belleghem et al., 2017). In comparisons between parapatric colour pattern races of both *H. erato* and *H. melpomene*, sharp *F*_ST_ peaks are present near the major colour pattern loci, suggesting both strong divergent selection and reduced gene flow (Figure S4). Additionally, increased *F*_ST_ values can be observed on the Z chromosome in comparisons between populations with assortative mating and hybrid inviability and sterility (Figures 3 and S4). However, *F*_ST_ is influenced by effective population sizes (Box 2). It is therefore problematic to obtain insights about selection or migration when comparing genomic regions with different effective population sizes, such as the Z chromosome and autosomes. Given equal numbers of breeding males and females, the Z chromosome is expected to have an effective population size three‐quarters that of the autosomes. Smaller population size and the resulting lower nucleotide diversity on the Z chromosome may therefore partly explain inflated *F*_ST_ estimates on the Z chromosome.

In contrast, *d*_XY_ values on the Z chromosome tend to be similar to or slightly lower than the average values on the autosomes in most species comparisons of the *H. erato* and *H. melpomene* clade (Figures 3 and S5). Under equilibrium conditions, *d*_XY_ on the Z chromosome is expected to be three‐quarters that of the autosomes at the time of the split. As time progresses and differences between populations accumulate, the proportion of the coalescent that is affected by the ancestral population size will become smaller and the ratio of *d*_XY_ on the Z to *d*_XY_ on the autosomes is expected to move towards one (Box 2). Estimating the exact split time is difficult, and finding the expected absolute divergence for the Z chromosome compared to the autosomes is complicated (Patterson, Richter, Gnerre, Lander, & Reich, 2006). However, in contrast to the expectation that the ratio of *d*_XY_ will move towards one, *d*_XY_ on the Z chromosome is higher than on the autosomes for *H. e. cyrbia*–*H. himera* and *H. e. venus*–*H. e. chestertonii* comparisons (Figure 3).

Finally, by subtracting an estimate of diversity in the ancestral population from the absolute divergence measure *d*_XY_, known as *d*_a_ (Nei & Li, 1979), we obtain an estimate of nucleotide differences that have accumulated since the time of split (Box 2). The *d*_a_ estimates show significantly higher divergence on the Z chromosome in the comparisons *H. himera*–*H. e. cyrbia*,* H. e. venus*–*H. e. chestertonii*, and in *H. melpomene*–*H. cydno* and *H. melpomene*–*H. timareta* (Figures 3 and S6). Overall, the increased *d*_XY_ in the *H. e. cyrbia* and *H. himera* and the *H. e. venus*–*H. e. chestertonii* comparisons and the higher *d*_a_ values on the Z chromosome relative to the autosomes appear to support a faster rate of divergence between *Heliconius* species pairs on the Z chromosome.

### 3.3 Population size changes affect the Z chromosome differently

**Figure 4:**
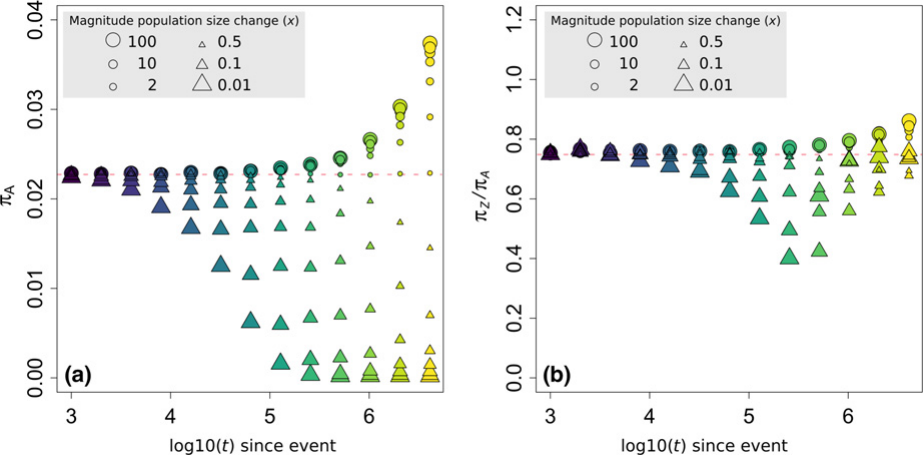
Simulated effect of population size change on nucleotide diversity on the autosomes (π_A_) (a) and the ratio of nucleotide diversity between the Z chromosome and autosomes (π_Z_/π_A_) (b). We simulated a single population that underwent a population size change of magnitude *x,* ranging from 0.01 to 100, at a certain moment backwards in time (*t*). Triangles indicate population size contractions, and circles indicate population size increase. Colours and *x*‐axis represent the time at which the population size change occurred in generations (log_10_(*t*), going from 1000 to 4,000,000 generations ago). Population size (*N*_e_) was 3e6 and 2.25e6 for the autosomes and Z chromosome, respectively. The colours and time at which population size change occurred correspond to colours in the simulations in Figure 7. The pink dashed lines indicate expectations under neutrality [Colour figure can be viewed at http://wileyonlinelibrary.com]

Apart from the overall difference in effective population size between the Z chromosome and autosomes, there are additional demographic factors that can contribute to differences in *F*_ST_, *d*_XY_ and *d*_a_ values between the Z chromosome and autosomes. Population size changes can alter the equilibrium expectation that Z‐linked diversity should be three‐quarters of autosomal diversity (Pool & Nielsen, 2007). To explore this, we performed coalescent simulations of sequences from populations that underwent a single size change in the past, varying the time and magnitude of this event (Figure 4). In these simulated populations, the decrease in nucleotide diversity that follows population contraction occurs much faster than the increase in diversity that follows an expansion of the same magnitude (Figure 4a). This is because an increase in diversity requires mutation accumulation, whereas drift can more rapidly remove variation to reach a new equilibrium. Additionally, population size changes have proportionately stronger effects on diversity on the Z chromosome compared to the autosomes (Figure 4b). This results from populations with a smaller effective population size, such as the Z chromosome, converging faster to their new equilibrium after a population size change (Pool & Nielsen, 2007).

**Figure 5:**
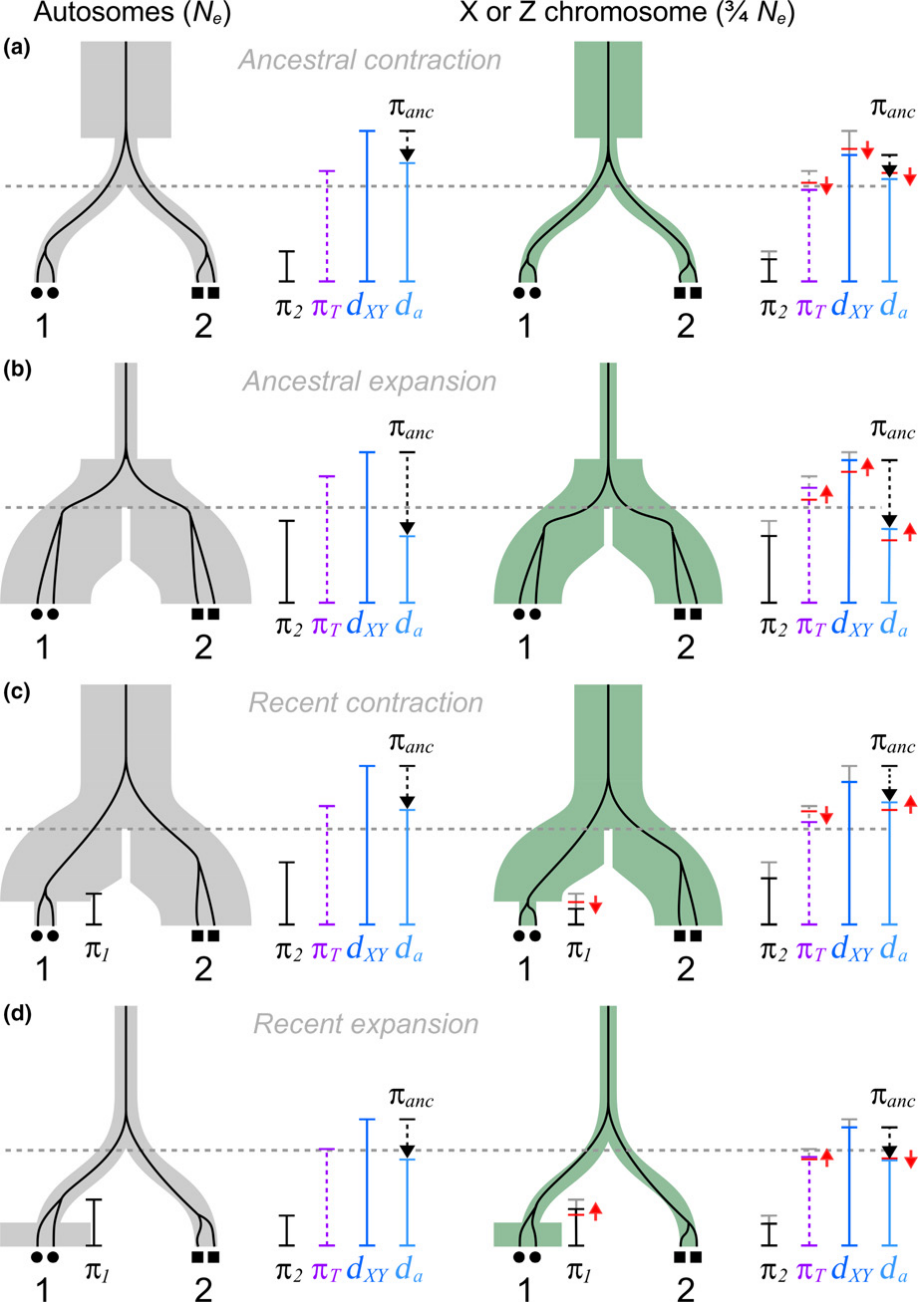
The effect of population size change on the coalescent and measures of diversity and divergence. The branches represent two populations, 1 and 2, that have split at a certain time (grey dashed line). This branching event occurs on two chromosomes that have a different population size, such as the autosomes (grey) and X chromosome (green). The black lines show the coalescent of two alleles in each population. This coalescence process is influenced by the split time as well as the population size (see Box 2). Moreover, red arrows show the disproportionate effect of population size change on diversity and divergence measures on the sex chromosome (or populations with a smaller size). Note that the effect size will depend on the magnitude as well as the timing of population size change (see Figure 4). Exact expectations, including more complex size changes, can be calculated as demonstrated in Pool and Nielsen (2007) [Colour figure can be viewed at http://wileyonlinelibrary.com]

The result of population size change differently affecting the Z chromosome is that divergence measures are also differentially affected by population size change on the Z chromosome compared to the autosomes. The Z chromosome to autosome (Z/A) diversity ratio will be larger than expected in populations that experienced a recent expansion and smaller than expected in those that experienced a recent contraction (Figure 4b). Therefore, in pairwise comparisons, if population size change occurred in the ancestral population before the two populations split, it would alter the ancestral Z/A diversity ratio and therefore confound comparisons of divergence between Z and autosomes using either relative or absolute measures of divergence, as all are influenced by ancestral diversity (Figure 5). By contrast, if population size change occurred in one or both daughter populations after the split, it would affect the relative measures of divergence *F*_ST_ and *d*_a_, but not absolute divergence (*d*_XY_), which is only dependent on ancestral diversity and not on diversity within each population. The effect size will depend on the timing and magnitude of the population size change. In our simulations, Z/A diversity ratios ranged from 0.40 to 0.86 under the most extreme simulated population size changes, compared to the expected diversity ratio of 0.75 under equilibrium expectations (Figure 4b). All the simulations were run for timescales relevant to *Heliconius* divergence and, therefore, demonstrate that a return to equilibrium values is unlikely after a population increase during the history of these species. In particular, a return to equilibrium Z/A diversity ratios after population size increase can be slow and long‐lasting during the evolutionary history of a population.

### 3.4 Demography and its influence on Z/A diversity ratios in *Heliconius*

**Figure 6:**
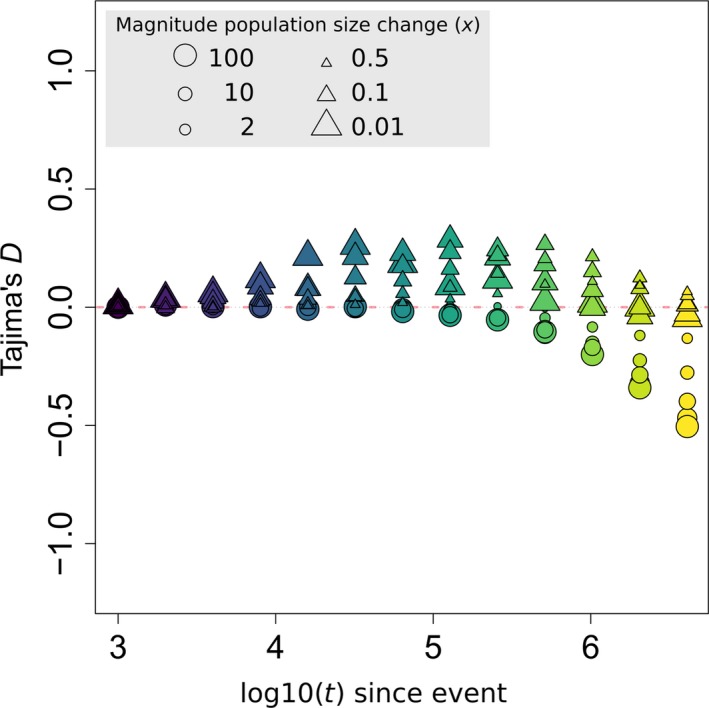
Simulated effect of population size change on Tajima's *D* values. We simulated a single population that underwent a population size change of magnitude *x,* ranging from 0.01 to 100, at a certain moment backwards in time (*t*). Triangles indicate population size contractions, and circles indicate population size increase. Colours and *x*‐axis represent the time at which the population size change occurred in generations (log_10_(*t*), going from 1000 to 4,000,000 generations ago) with population size (*N*_e_) equal to 3e6 and 2.25e6 for the autosomes and Z chromosome, respectively. The colours and time at which population size change occurred correspond to colours in the simulations in Figure 7. The pink dashed line indicates expectations under neutrality [Colour figure can be viewed at http://wileyonlinelibrary.com]

**Figure 7:**
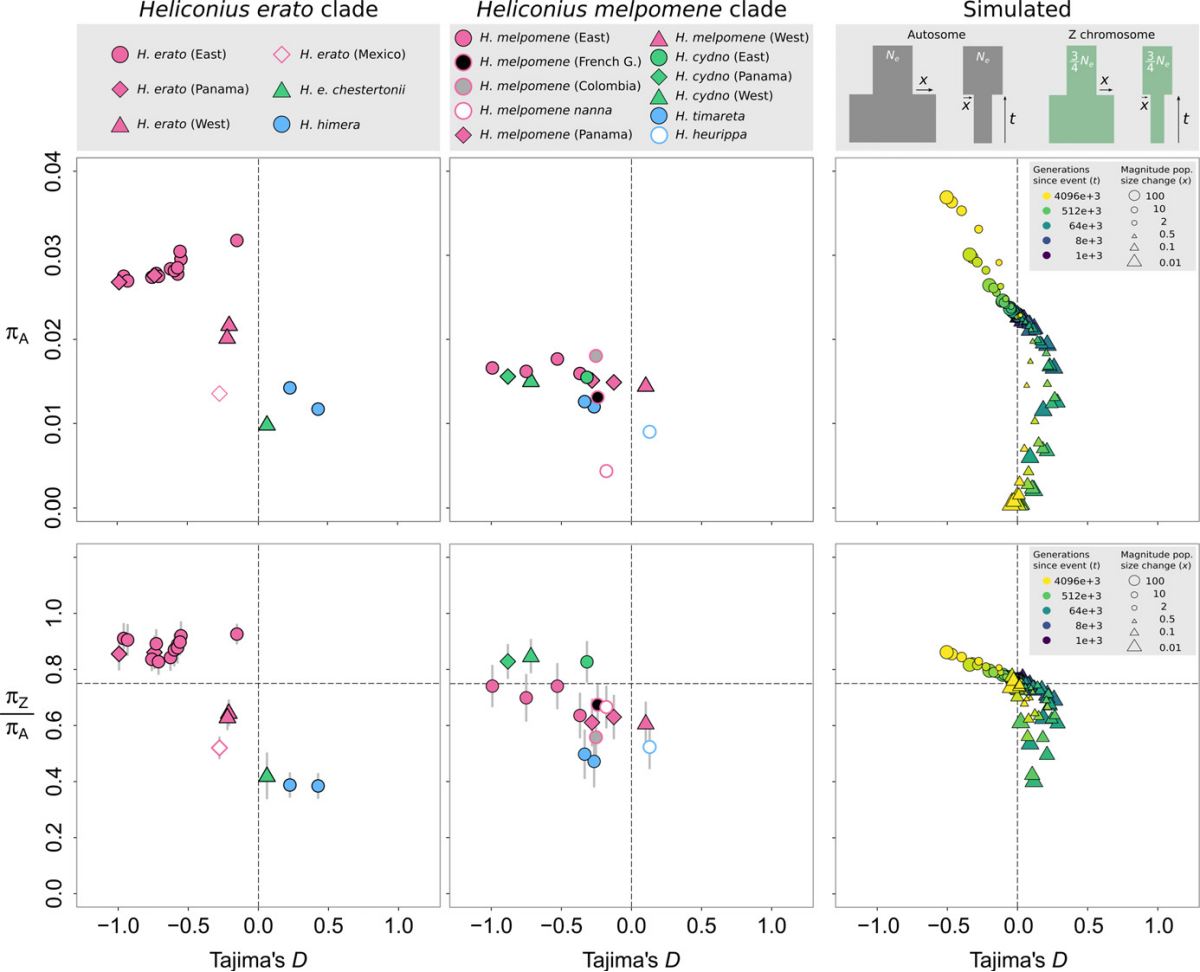
Relation between Tajima's *D*, average nucleotide diversity on the autosomes (π_A_) (upper panels) and the ratio of nucleotide diversity between the Z chromosome and autosomes (π_Z_/π_A_) (lower panels) for populations of the *Heliconius erato* and *Heliconius melpomene* clade and simulated data. Points represent average nucleotide diversity measures obtained from autosomes (π_A_) and the Z chromosome (π_Z_) in 50‐kb windows. Grey vertical bars represent 95% confidence intervals estimated from block‐jackknifing (note that these are too small to see in the Tajima's *D* versus π_A_ plots). Schematics in the upper right panel represent the simulated population size changes. We simulated a single population that underwent a population size change of magnitude *x,* ranging from 0.01 to 100 (right panels), with population size change occurring between 1000 and 4,000,000 generations (t) ago (colours). Triangles indicate population size contractions, and circles indicate population size increase. Population size (*N*_e_) was 3e6 and 2.25e6 for the autosomes and Z chromosome, respectively. The dashed lines indicate expectations under neutrality [Colour figure can be viewed at http://wileyonlinelibrary.com]

To explore how population size changes might have affected Z/A diversity ratios and thus Z/A divergence comparisons within *Heliconius* clades, we used the behaviour of Tajima's *D* as a way to assess likely population size changes within species. Tajima's *D* is a population genetic measure commonly used to detect whether a locus is evolving neutrally in a population (Tajima, 1989). At a genomewide scale, negative values reflect population size expansion, whereas positive values can reflect population size decrease or population subdivision. Due to the different response of Tajima's *D* to population size increase and decrease, Tajima's *D* can give an indication of population size changes and their effect on nucleotide diversity. As the simulations show, negative Tajima's *D* values (population size increase) are correlated with increased nucleotide diversity, whereas positive Tajima's *D* values (population size decrease) are correlated with reduced nucleotide diversity (Figure 6). As with the Z/A diversity ratio, the timescale of the influence of population size change on Tajima's *D* values is different for population expansion versus population contraction, as a population that has contracted returns to equilibrium faster than one that has expanded. Moreover, because smaller populations respond faster to such population size changes, the Tajima's *D* values are also expected to be correlated with Z/A diversity ratios. Although this results in a complex relationship (Figure 7), *H. erato* and *H. melpomene* clade populations that showed more negative Tajima's *D* values (~population size increase) all had higher nucleotide diversity (π) values, as well as higher Z/A diversity ratios (Figure 7). Z/A diversity ratios ranged from 0.38 to 0.93 in the *H. erato* clade and from 0.47 to 0.85 in the *H. melpomene* clade, similar to the range of values obtained in the simulations (Figure 4b and 7). It should be noted that multiple population size change events (e.g., population size expansion followed by a bottleneck), continuous increase and different durations of population size changes would further complicate the relation between Tajima's *D* estimates and the expected nucleotide diversity as well as the Z/A diversity ratio. Potentially, this also explains the more extreme negative Tajima's *D* and Z/A diversity ratios in several of the *H. erato* and *H. melpomene* clade populations compared to our simulated scenarios (Figure 7). Nevertheless, Tajima's *D* estimates for the *H. erato* and *H. melpomene* clade do capture the average demographic history as inferred using the PSMC method. We find more negative Tajima's *D* values for populations that have undergone continuous population size increase and higher Tajima's *D* values for populations that have undergone steep population declines (Figures 2 and 7). Therefore, the patterns among these *Heliconius* populations suggest that differences in nucleotide diversity as well as differences in the Z/A diversity ratios are likely driven at least in part by population size changes. Given that samples assigned to a population were collected in close proximity, it is unlikely that estimated Tajima's *D* values are influenced by hidden population structure in the data (which could result in positive Tajima's *D* values).

**Figure 8:**
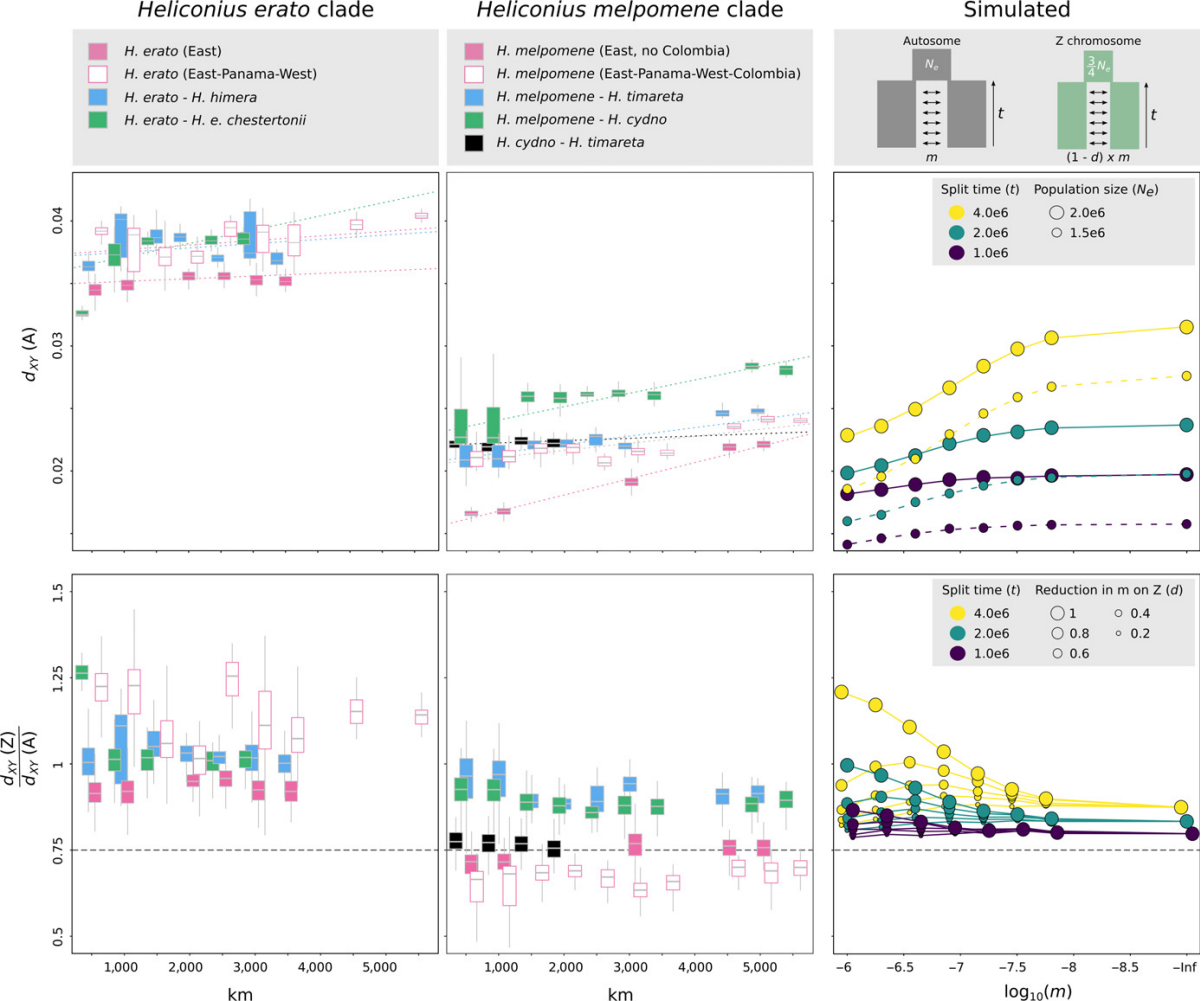
Relation between pairwise distance (km), autosomal *d*_XY_ (a) (upper panels) and the ratio of *d*_XY_ between the Z chromosome and autosomes (*d*_XY_ (Z)/*d*_XY_ (A)) for populations of the *Heliconius erato* and *Heliconius melpomene* clade and simulated data. Boxplots represent pairwise measures over 500‐km bins. Schematics in the upper right panel represent the simulated populations with different rates of migration (*m*) expressed as a proportion of the effective population size. We considered pairs of populations that split between 1 and 2 million generations ago (colours) and were connected through migration (*m*) ranging from 0 to 1e‐6 and for which migration was reduced with a factor *d* on the Z chromosome (size of circles). Population size (*N*_e_) was equal to 2e6 and 1.5e6 for the autosomes and Z chromosome, respectively [Colour figure can be viewed at http://wileyonlinelibrary.com]

Broad patterns of nucleotide diversity can also give insights into the general demography of the studied species. Higher nucleotide diversity in the *H. erato* clade as compared to the *H. melpomene* clade is consistent with the generally greater abundance of *H. erato* observed in nature (Mallet, Jiggins, & McMillan, 1998) (Figure 7). These population size differences likely also explain the large differences in absolute divergence levels among the *H. erato* clade populations compared to the *H. melpomene* clade populations (Figure 8). Absolute divergence between *H. melpomene* and *H. timareta* or *H. cydno*, for instance, is smaller than within‐population diversity of most *H. erato* populations (Figure 7), despite the former pairs being clearly distinct species.

While changes in population size can have strong effects on measures of sequence divergence, jointly considering patterns of variation on the Z chromosome and autosome can give further insights into the evolutionary history. Among *H. erato* populations from east of the Andes that show little differentiation, *d*_XY_(Z)/*d*_XY_(A) ratios are above the 0.75 ratio that would be expected immediately after the populations split (0.91 ± 0.11) (Figure 8) and there is also increased Z/A nucleotide diversity (Figure 7). This likely resulted from population size increase in the ancestral population. Similarly, if this population size increase occurred before the divergence of *H. himera* and *H. e. chestertonii* from *H. erato,* this could have contributed to elevated *d*_XY_(Z)/*d*_XY_(A) in these comparisons (Figure 7). In contrast, the *d*_XY_(Z)/*d*_XY_(A) ratios among *H. melpomene* from east of the Andes are closer to the 0.75 (Figure 8). While comparisons between *H. melpomene* populations east and west of the Andes, Panama and Colombia show deeper divergence, their *d*_XY_(Z)/*d*_XY_(A) ratios are much lower, consistent with a population size decrease deeper in the ancestry of *H. melpomene*. Finally, the lower *d*_XY_(Z)/*d*_XY_(A) ratios in *H. cydno*–*H. timareta* comparisons relative to the *H. melpomene–H. cydno* and *H. melpomene–H. timareta* comparisons potentially suggest a population contraction of the ancestral population of *H. cydno* and *H. timareta*, but after they split from *H. melpomene*.

Within the *H. erato* clade, nucleotide diversity as well as Z/A diversity ratios were distinctly higher in populations from east of the Andes and Panama and lower in the *H. e. chestertonii* and *H. himera* populations (Figure 7). These populations shared a common ancestor, so differences in nucleotide diversity likely result from population size changes that occurred after divergence and thus confound the relative *F*_ST_ and *d*_a_ divergence measures. Although absolute divergence *d*_XY_ is clearly higher between *H. erato* and *H. himera* or *H. e. chestertonii* than among *H. erato* populations east of the Andes (Figure 8), a population size decrease in *H. himera* and *H. e. chestertonii* may inflate the *F*_ST_ and *d*_a_ estimates when comparing these populations to geographically abutting *H. erato* populations (Figure 3). Additionally, any population size changes that occurred before the split of *H. himera* from *H. erato* and *H. e. chestertonii* from *H. erato* may have affected current *d*_XY_ estimates. Importantly, if such demographic changes differently affected the ancestor of *H. himera* as compared to the ancestor of *H. e. chestertonii,* the *d*_XY_ values may not necessarily reflect different degrees or stages of the speciation process. This difficulty may also apply when comparing divergence between *H. melpomene* and *H. timareta* and between *H. melpomene* and *H. cydno*.

### 3.5 Sex‐linked incompatibilities increase Z/A absolute divergence ratio

Despite the difficulties in directly comparing divergence on sex chromosomes and autosomes, it may be possible to detect enhanced barriers to migration on sex chromosomes (i.e., reduced *effective* migration) by comparing population pairs with different levels of *absolute* migration due to physical isolation, but that otherwise share the same common history. This can be achieved by comparing pairs of populations from the same two species that differ in their extent of geographic isolation. Indeed, previous analyses of sympatric and allopatric populations of *H. melpomene*,* H. cydno* and *H. timareta*, based on shared derived alleles (i.e., the ABBA‐BABA test), found evidence of extensive gene flow between the species in sympatry, but with a strong reduction on the Z chromosome (Martin et al., 2013). Here, we instead use our broad sampling scheme to investigate how patterns of sequence divergence differ with differing levels of geographic separation, and ask whether this signal can detect reduced effective migration on the Z chromosome.

Among *H. erato* and *H. melpomene* clade populations, absolute divergence generally increases with increased distance between population pairs (Figure 8). This trend is strongest for population comparisons that are less obstructed by geographic barriers, such as among *H. erato* (Mantel's test: *R* ^2^ = .18; *p* = .012) and *H. melpomene* (excluding Colombia; Mantel's test: *R* ^2^ = .95; *p* = .001) populations from east of the Andes. As expected, the correlation between distance and absolute divergence is reduced by geographical barriers, such as when comparing *H. erato* (Mantel's test: *R* ^2^ = .15; *p* = .019) and *H. melpomene* (Mantel's test: *R* ^2^ = .55; *p* = .001) populations from Panama, east of the Andes and west of the Andes. We also observed a significant trend of increased absolute divergence with distance between populations of *H. erato*–*H. e. chestertonii* (Mantel's test: *R* ^2^ = .44; *p* = .001), *H. melpomene*–*H. cydno* (Mantel's test: *R* ^2^ = .69; *p* = .001) and *H. melpomene*–*H. timareta* (Mantel's test: *R* ^2^ = .56; *p* = .001). This is consistent with gene flow among these species pairs where they are in contact. In contrast, no significant trend between absolute divergence and distance was observed between *H. erato*–*H. himera* and *H. cydno*–*H. timareta*, suggesting that these species pairs may be more strongly isolated.

Simulations show that if distance is considered a proxy for migration, reduced rates of admixture on the Z chromosome may become apparent as increased Z/A absolute divergence (*d*_XY_(Z)/*d*_XY_(A)) ratios over short distances, with the ratio decreasing between pairs that are geographically more isolated (Figure 8). As the effective rate of migration is reduced on the Z chromosome relative to autosomes, the *d*_XY_(Z)/*d*_XY_(A) ratio increases, and this increase is most pronounced when overall migration rates are high. This relationship can be explained by the absolute difference in effective migration on the Z chromosome compared to the autosomes becoming smaller as overall migration decreases. While overall *d*_XY_(Z)/*d*_XY_(A) ratios may be influenced by ancestral population size changes, the trend should be independent from population size changes that occurred after the populations split. Our widespread sampling of both *Heliconius* clades therefore allowed us to test for reduced effective migration on the Z chromosome.

First, we examined the *d*_XY_(Z)/*d*_XY_(A) ratios and its relationship to geographic distance. If rates of admixture between *Heliconius* populations are similar on the Z chromosome compared to the autosomes, we would not expect any relation between distance and *d*_XY_(Z)/*d*_XY_(A) ratios. In contrast, we observed increased *d*_XY_(Z)/*d*_XY_(A) ratios among geographically more closely located population pairs for *H. melpomene*–*H. timareta* (Mantel's test: *R* ^2^ = .25; *p* = .004) and *H. melpomene*–*H. cydno* (Mantel's test: *R* ^2^ = .35; *p* = .001) comparisons (Figure 8). Similarly, a tendency for increased *d*_XY_(Z)/*d*_XY_(A) ratios between *H. erato*–*H. e. chestertonii* was observed among the geographically closest comparisons, although this was not significant (Mantel's test: R^2^ = .26; *p* = .06). Finding this trend, however, can be obscured by geographic barriers that would reduce the relation between distance and admixture. For instance, *H. e. chestertonii* comes into close contact with *H. e. venus* west of the Andes, but is geographically isolated from relatively closely located *H. erato* populations east of the Andes. Similarly, PCA of the *H. melpomene* populations indicates splits between populations east of the Andes, which may reflect additional geographic barriers that do not correlate linearly with distance (Figure 1).

**Figure 9:**
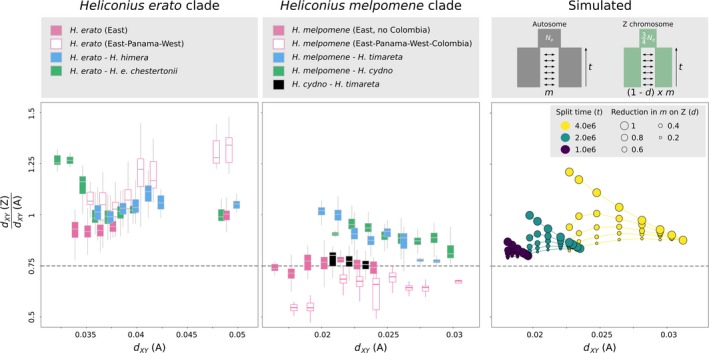
Relation between pairwise autosomal *d*_XY_ (a) and the ratio of *d*_XY_ between the Z chromosome and autosomes (*d*_XY_ (Z)/*d*_XY_ (A)) for populations of the *Heliconius erato* and *Heliconius melpomene* clade and simulated data. Boxplots represent pairwise measures over 1.25e‐3 *d*_XY_ bins. Schematics in the upper right panel represent the simulated populations with different rates of migration (*m*) expressed as a proportion of the effective population size. We considered pairs of populations that split between 1 and 2 million generations ago (colours) and were connected through migration (*m*) ranging from 0 to 1e‐6 and for which migration was reduced with a factor *d* on the Z chromosome (size of circles). Population size (*N*_e_) was equal to 2e6 and 1.5e6 for the autosomes and Z chromosome, respectively [Colour figure can be viewed at http://wileyonlinelibrary.com]

Next, to account for geographic barriers, we also carried out a similar comparison using absolute divergence on the autosomes instead of geographic distance, which might reflect a more direct relationship with migration. Using the absolute divergence on the autosomes as a proxy for gene flow, we find a pattern of increased Z/A divergence ratios for species pairs with known postzygotic reproductive barriers (Figure 9). Z/A divergence ratios are significantly higher between population pairs with lower divergence values on the autosomes in *H. melpomene*–*H. timareta* (Mantel's test: *R* ^2^ = .70; *p* = .001), *H. melpomene–H. cydno* (Mantel's test: *R* ^2^ = 0.64; *p* = .001) and *H. erato*–*H. e. chestertonii* (Mantel's test: *R* ^2^ = .51; *p* = .007) comparisons, but not in *H. timareta*–*H. cydno* and *H. erato*–*H. himera* comparisons. This is consistent with crosses showing that the former and not the latter pairs experience hybrid sterility and Haldane's rule (McMillan et al., 1997; Merrill et al., 2012; Muñoz et al., 2010; Naisbit et al., 2002; Salazar et al., 2005; Sánchez et al., 2015) and also agrees with the previous observation of reduced shared variation between *H. melpomene* and both *H. timareta* and *H. cydno* on the Z chromosome (Martin et al., 2013). Note that our simulations suggest that to explain the trend observed in our data, there must be a very strong reduction in migration on the Z chromosome relative to the autosomes (~60% or greater).

### 3.6 Alternative factors affecting Z/A diversity ratios in Heliconius

Factors other than population size change could result in deviations from the expected Z/A diversity ratio (Box 1). In *Heliconius*, there is no empirical data on sex chromosome‐biased mutation rates. While higher mutation rates on the Z could explain increased Z/A diversity ratios and increased rates of divergence (Kirkpatrick & Hall, 2004; Sayres & Makova, 2011; Vicoso & Charlesworth, 2006), it is unlikely that closely related populations would differ in their mutation rate and that this could explain the observed variation in Z/A diversity ratios among the *Heliconius* populations. Alternatively, in *Heliconius,* male‐biased sex ratios have been reported in the field, which could result in increased Z/A diversity ratios. However, it has been argued that these male‐biased sex ratios are most likely explained by differences in behaviour, resulting in male‐biased captures rather than effective sex ratio differences (Jiggins, 2017). Nonetheless, a *Heliconius* characteristic that could potentially amplify sex ratio biases is that *H. erato* and *H. melpomene* clade populations are characterized by contrasting pupal‐mating and adult‐mating strategies, respectively (Beltrán, Jiggins, Brower, Bermingham, & Mallet, 2007; Gilbert, 1976). Pupal maters are largely monandrous (females mate only once), whereas adult maters are polyandrous (Walters, Stafford, Hardcastle, & Jiggins, 2012). Such differences in mating system could potentially result in increased variance of male reproductive success and decreased Z/A diversity ratios for monandrous mating systems (Charlesworth, 2001). However, the frequency of remating in polyandrous *Heliconius* species is estimated to be only 25–30% higher than in monandrous species (Walters et al., 2012) and adult mating is likely still prevalent in presumed pupal‐mating species (Thurman, Brodie, Evans, & McMillan, 2018). Correspondingly, we did not find any clear difference in Z/A diversity ratios between the pupal‐mating *H. erato* and adult‐mating *H. melpomene* clade populations (Figure 7). Finally, it has been suggested that effective recombination may be higher for the Z chromosome when recombination is completely absent in females (Charlesworth, 2012). This is because the Z chromosomes spend two‐thirds of their time in recombining males, whereas autosomes only spend half of their time in recombining males. This could lead to less of a reduction in diversity on the Z compared to autosomes than the 25% null expectation. However, this cannot explain the correspondence of Tajima's *D* and the PSMC inferences and the observed Z/A diversity ratios. Similarly, the pattern of increased Z/A divergence that results from reduced admixture on the Z in population comparisons of geographically closely located *Heliconius* species should not be affected by sex ratio or recombination and mutation biases. Overall, in *Heliconius*, the largest influence on variation in Z/A diversity ratios is likely to be demographic changes.

### 3.7 Consequences for other study systems

Extensive genomic sampling is available for a number of other natural systems that have recently diverged, particularly for birds that also have ZW sex chromosomes, such as flycatchers (Ellegren et al., 2012), crows (Poelstra et al., 2014) and Darwin's finches (Lamichhaney et al., 2015). In these systems, increased coalescence rates (~lineage sorting) on the Z and/or W chromosome have been accredited to the smaller effective population sizes of the sex chromosome. However, it remains unclear whether elevated measures of divergence could indicate elevated rates of between species divergence on the sex chromosomes, resulting from increased mutation or reduced admixture.

In the adaptive radiation of Darwin's finches, there is no evidence for Haldane's rule nor for reduced viability of hybrids due to postmating incompatibilities (Grant & Grant, 1992) and the maintenance of isolating barriers is best explained as resulting from ecological selection and assortative mating (Grant & Grant, 2008). In crows, the divergence between hooded and carrion crows seems to be solely associated with colour‐mediated assortative mating even in the apparent absence of ecological selection (Poelstra et al., 2014; Randler, 2007). The populations of both Darwin's finches and crows can be characterized by distinct demographic histories (Lamichhaney et al., 2015; Vijay et al., 2016). Therefore, in these species, deviations in divergence measures from neutral expectation on the Z chromosome are potentially also explained by demography. In the divergence of pied and collared flycatchers, species recognition and species‐specific male plumage traits are Z‐linked (Saether et al., 2007) and female hybrids are completely sterile compared to only low levels of reduced fertility in males (Veen et al., 2001). In agreement with the large‐X effect and disjunct rates of admixture between the sex chromosomes and autosomes, genome scans have found signals of increased relative divergence on the Z and W chromosomes (Ellegren et al., 2012; Smeds et al., 2015). The demographic history of these populations is, however, characterized by a severe decrease in population size since their divergence (Nadachowska‐Brzyska et al., 2013). In particular, for the W chromosome, the reported excessive decrease in diversity and the high values of relative divergence can thus likely be partly explained by demography (Smeds et al., 2015). However, the excess of rare alleles (~negative Tajima's *D*) on the W chromosome does contrast with these inferred demographic histories and provides support that the reduced diversity and increased *F*_ST_ measures result from selection (Smeds et al., 2015).

## 4 CONCLUSION

The disproportionate role of sex chromosomes during speciation has been well documented based on genetic analysis. However, it is less clear how this influences patterns of divergence in natural populations. In *Heliconius*, we find much of the observed increased absolute divergence on the Z chromosome relative to neutral expectation can be explained by population size changes. This cautions against highlighting increased sex chromosome divergence alone as evidence for a disproportionate role in species incompatibilities or as evidence for faster‐X evolution. Although relative measures of divergence are most prone to demographic changes, absolute divergence measures can also be strongly influenced by population size changes. Absolute measures do not therefore provide a solution to the problems inherent in using relative measures to compare patterns of divergence across genomes (Cruickshank & Hahn, 2014). Despite these difficulties, we do find patterns consistent with decreased effective migration on the Z for species pairs with known postzygotic reproductive barriers, in agreement with hybrid sterility and inviability being linked to the Z chromosome in these cases (Jiggins, Linares, et al., 2001; Naisbit et al., 2002; Salazar et al., 2005; Sánchez et al., 2015). Successfully disentangling the influence of a large‐X effect and faster‐X evolution on relative rates of divergence will require modelling of the demographic history of each population, including changes that may have occurred before the split of the populations. Such modelling would allow us to better contrast (i) expected within‐population Z/A diversity ratios with hypotheses of increased mutation rates, selective sweeps, background selection and mating system and (ii) expected between population Z/A divergence ratios with hypotheses of increased mutation rates or adaptive divergence on the Z chromosome. Additionally, our strategy of contrasting *d*_XY_(Z)/*d*_XY_(A) ratios with geographic distance provides opportunities for testing reduced admixture between sex chromosomes in systems for which tree‐based approaches and/or crossing experiments are unfeasible.

## DATA ACCESSIBILITY

Genome assemblies are available on lepbase.org. Sequencing reads are deposited in the Sequence Read Archive (SRA). See Tables S1 and S2 for accession numbers.

## AUTHOR CONTRIBUTIONS

S.V.B., M.B., B.A.C., C.D.J. and S.H.M. conceived of the study. S.V.B., M.B. and S.H.M. analyzed the data. S.V.B., M.B., W.O.M., B.A.C., C.D.J. and S.H.M. wrote the paper. R.P. and C.S. provided samples. All authors discussed the results and contributed to the final manuscript.

## References

[mec14560-bib-0001] Arias, C. F. , Muñoz, A. G. , Jiggins, C. D. , Mavarez, J. , Bermingham, E. , & Linares, M. (2008). A hybrid zone provides evidence for incipient ecological speciation in *Heliconius* butterflies. Molecular Ecology, 17, 4699–4712. 10.1111/j.1365-294X.2008.03934.x 18828780

[mec14560-bib-0002] Arias, C. F. , Salazar, C. , Rosales, C. , Kronforst, M. R. , Linares, M. , Bermingham, E. , & McMillan, W. O. (2014). Phylogeography of *Heliconius cydno* and its closest relatives: Disentangling their origin and diversification. Molecular Ecology, 23, 4137–4152. 10.1111/mec.12844 24962067

[mec14560-bib-0003] Beltrán, M. , Jiggins, C. , Brower, A. , Bermingham, E. , & Mallet, J. (2007). Do pollen feeding and pupal‐mating have a single origin in *Heliconius* butterflies? Inferences from multilocus sequence data. Biological Journal of the Linnean Society, 92, 221–239. 10.1111/(ISSN)1095-8312

[mec14560-bib-0004] Charlesworth, B. (1998). Measures of divergence between populations and the effect of forces that reduce variability. Molecular Biology and Evolution, 15, 538–543. 10.1093/oxfordjournals.molbev.a025953 9580982

[mec14560-bib-0005] Charlesworth, B. (2001). The effect of life‐history and mode of inheritance on neutral genetic variability. Genetic Research, 77, 153–166. 10.1017/s0016672301004979 11355571

[mec14560-bib-0006] Charlesworth, B. (2012). The role of background selection in shaping patterns of molecular evolution and variation: Evidence from variability on the *Drosophila X* chromosome. Genetics, 191, 233–246. 10.1534/genetics.111.138073 22377629 PMC3338263

[mec14560-bib-0007] Charlesworth, B. , Coyne, J. A. , & Barton, N. H. (1987). The relative rates of evolution of sex chromosomes and autosomes. American Naturalist, 130, 113–146. 10.1086/284701

[mec14560-bib-0008] Counterman, B. A. , Ortíz‐Barrientos, D. , & Noor, M. A. F. (2004). Using comparative genomic data to test for fast‐X evolution. Evolution; International Journal of Organic Evolution, 58, 656–660. 10.1111/j.0014-3820.2004.tb01688.x 15119449

[mec14560-bib-0009] Coyne, J. , & Orr, H. (1989). Two rules of speciation In Otte D., & Endler J. (Eds.), Speciation and its consequences (pp. 180–207). Sunderland, MA, USA: Sinauer Associates Inc.

[mec14560-bib-0010] Coyne, J. A. , & Orr, H. A. (2004). Speciation. Sunderland, MA, USA: Sinauer Associates.

[mec14560-bib-0011] Cruickshank, T. E. , & Hahn, M. W. (2014). Reanalysis suggests that genomic islands of speciation are due to reduced diversity, not reduced gene flow. Molecular Ecology, 23, 3133–3157. 10.1111/mec.12796 24845075

[mec14560-bib-0012] Dasmahapatra, K. K. , Walters, J. R. , Briscoe, A. D. , Davey, J. W. , Whibley, A. , Nadeau, N. J. , … Salazar, C. (2012). Butterfly genome reveals promiscuous exchange of mimicry adaptations among species. Nature, 487, 94–98. 10.1038/nature11041 22722851 PMC3398145

[mec14560-bib-0013] Davey, J. W. , Barker, S. L. , Rastas, P. M. , Pinharanda, A. , Martin, S. H. , Durbin, R. , … Jiggins, C. D. (2017). No evidence for maintenance of a sympatric *Heliconius* species barrier by chromosomal inversions. Evolution Letters, 1, 138–154. 10.1002/evl3.12 30283645 PMC6122123

[mec14560-bib-0014] Davey, J. W. , Chouteau, M. , Barker, S. L. , Maroja, L. , Baxter, S. W. , Simpson, F. , … Jiggins, C. D. (2016). Major improvements to the *Heliconius melpomene* genome assembly used to confirm 10 chromosome fusion events in 6 million years of butterfly evolution. G3, 6, 695–708. 10.1534/g3.115.023655 26772750 PMC4777131

[mec14560-bib-0015] De Mita, S. , & Siol, M. (2012). EggLib: Processing, analysis and simulation tools for population genetics and genomics. BMC Genetics, 13, 27 10.1186/1471-2156-13-27 22494792 PMC3350404

[mec14560-bib-0016] Dobzhansky, T. (1935). Studies on hybrid sterility. II. Localization of factors in *Drosophila pseudoobscura* hybrids. Genetics, 21, 113–135. 10.1093/genetics/21.2.113 PMC1208664 17246786

[mec14560-bib-0017] Ellegren, H. , Smeds, L. , Burri, R. , Olason, P. I. , Backström, N. , Kawakami, T. , … Uebbing, S. (2012). The genomic landscape of species divergence in *Ficedula* flycatchers. Nature, 491, 756–760. 23103876 10.1038/nature11584

[mec14560-bib-0018] Ewing, G. , & Hermisson, J. (2010). MSMS: A coalescent simulation program including recombination, demographic structure and selection at a single locus. Bioinformatics (Oxford, England), 26, 2064–2065. 10.1093/bioinformatics/btq322 PMC2916717 20591904

[mec14560-bib-0019] Flanagan, N. S. , Tobler, A. , Davison, A. , Pybus, O. G. , Kapan, D. D. , Planas, S. , … McMillan, W. O. (2004). Historical demography of Müllerian mimicry in the neotropical *Heliconius* butterflies. Proceedings of the National Academy of Sciences of the United States of America, 101, 9704–9709. 10.1073/pnas.0306243101 15210977 PMC470739

[mec14560-bib-0020] Frank, S. A. (1991). Divergence of meiotic drive‐suppression systems as an explanation for sex‐biased hybrid sterility and inviability. Evolution, 45, 262–267. 28567880 10.1111/j.1558-5646.1991.tb04401.x

[mec14560-bib-0021] Gilbert, L. E. (1976). Postmating female odor in *Heliconius* butterflies: A male‐contributed antiaphrodisiac? Science, 193, 420–422. 935877 10.1126/science.935877

[mec14560-bib-0022] Gillespie, J. H. , & Langley, C. H. (1979). Are evolutionary rates really variable? Journal of Molecular Evolution, 13, 27–34. 10.1007/BF01732751 458870

[mec14560-bib-0023] Grant, P. R. , & Grant, B. R. (1992). Hybridization of bird species. Science, 256, 193–197. 10.1126/science.256.5054.193 17744718

[mec14560-bib-0024] Grant, P. R. , & Grant, B. R. (2008). Pedigrees, assortative mating and speciation in Darwin's finches. Proceedings of the Royal Society B: Biological Sciences, 275, 661–668. 10.1098/rspb.2007.0898 18211884 PMC2596835

[mec14560-bib-0025] Haldane, J. B. S. (1922). Sex ratio and unisexual sterility in hybrid animals. Journal of Genetics, 12, 101–109. 10.1007/BF02983075

[mec14560-bib-0026] Hudson, R. R. , Slatkin, M. , & Maddison, W. P. (1992). Estimation of levels of gene flow from DNA sequence data. Genetics, 132, 583–589. 1427045 10.1093/genetics/132.2.583 PMC1205159

[mec14560-bib-0027] Jablonka, E. , & Lamb, M. J. (1991). Sex chromosomes and speciation. Proceedings of the Royal Society B, 243, 203–208. 10.1098/rspb.1991.0032 1675798

[mec14560-bib-0028] Jiggins, C. D. (2017). The ecology and evolution of Heliconius butterflies, Oxford, UK: Oxford University Press.

[mec14560-bib-0029] Jiggins, C. D. , Linares, M. , Naisbit, R. E. , Salazar, C. , Yang, Z. H. , & Mallet, J. (2001). Sex‐linked hybrid sterility in a butterfly. Evolution, 55, 1631–1638. 10.1111/j.0014-3820.2001.tb00682.x 11580022

[mec14560-bib-0030] Jiggins, C. D. , Mcmillan, O. , Neukirchen, W. , Mallet, J. , & Nw, L. (1996). What can hybrid zones tell us about speciation? The case of *Heliconius erato* and *H. himera* (Lepidoptera: Nymphalidae). Biological Journal of the Linnean Society, 59, 221–242.

[mec14560-bib-0031] Jiggins, C. D. , Naisbit, R. E. , Coe, R. L. , & Mallet, J. (2001). Reproductive isolation caused by colour pattern mimicry. Nature, 411, 302–305. 10.1038/35077075 11357131

[mec14560-bib-0032] Johnson, N. A. , & Lachance, J. (2012). The genetics of sex chromosomes: Evolution and implications for hybrid incompatibility. Annals of the New York Academy of Sciences, 1256, E1–E22. 10.1111/j.1749-6632.2012.06748.x 23025408 PMC3509754

[mec14560-bib-0033] Keightley, P. D. , Pinharanda, A. , Ness, R. W. , Simpson, F. , Dasmahapatra, K. K. , Mallet, J. , … Jiggins, C. D. (2014). Estimation of the spontaneous mutation rate in *Heliconius melpomene* . Molecular Biology and Evolution, 32, 239–243. 25371432 10.1093/molbev/msu302 PMC4271535

[mec14560-bib-0034] Kirkpatrick, M. , & Hall, D. W. (2004). Male‐biased mutation, sex Linkage, and the rate of adaptive evolution. Evolution, 58, 437 10.1111/j.0014-3820.2004.tb01659.x 15068360

[mec14560-bib-0035] Kozak, K. M. , Wahlberg, N. , Neild, A. F. , Dasmahapatra, K. K. , Mallet, J. , & Jiggins, C. D. (2015). Multilocus species trees show the recent adaptive radiation of the mimetic *Heliconius* butterflies. Systematic Biology, 64, 505–524. 10.1093/sysbio/syv007 25634098 PMC4395847

[mec14560-bib-0036] Kronforst, M. R. , Hansen, M. E. B. , Crawford, N. G. , Gallant, J. R. , Zhang, W. , Kulathinal, R. J. , … Mullen, S. P. (2013). Hybridization reveals the evolving genomic architecture of speciation. Cell Reports, 5, 666–677. 10.1016/j.celrep.2013.09.042 24183670 PMC4388300

[mec14560-bib-0037] Künsch, H. R. (1989). The jackknife and the bootstrap for general stationary observations. Annals of Statistics, 17, 1217–1241. 10.1214/aos/1176347265

[mec14560-bib-0038] Lamichhaney, S. , Berglund, J. , Almén, M. S. , Maqbool, K. , Grabherr, M. , Martinez‐Barrio, A. , … Grant, B. R. (2015). Evolution of Darwin's finches and their beaks revealed by genome sequencing. Nature, 518, 371–375. 10.1038/nature14181 25686609

[mec14560-bib-0039] Lavretsky, P. , Dacosta, J. M. , Hernández‐Baños, B. E. , Engilis, A. , Sorenson, M. D. , & Peters, J. L. (2015). Speciation genomics and a role for the Z chromosome in the early stages of divergence between Mexican ducks and mallards. Molecular Ecology, 24, 5364–5378. 10.1111/mec.13402 26414437

[mec14560-bib-0040] Li, H. (2013). Aligning sequence reads, clone sequences and assembly contigs with BWA‐MEM. arXiv, 1303.3997v1.

[mec14560-bib-0041] Li, H. , & Durbin, R. (2011). Inference of human population history from individual whole‐genome sequences. Nature, 475, 493–496. 10.1038/nature10231 21753753 PMC3154645

[mec14560-bib-0042] Li, H. , Handsaker, B. , Wysoker, A. , Fennell, T. , Ruan, J. , Homer, N. , … Durbin, R. (2009). The Sequence Alignment/Map format and SAMtools. Bioinformatics, 25, 2078–2079. 10.1093/bioinformatics/btp352 19505943 PMC2723002

[mec14560-bib-0043] Lohse, K. , Chmelik, M. , Martin, S. H. , & Barton, N. H. (2016). Efficient strategies for calculating blockwise likelihoods under the coalescent. Genetics, 202, 775–786. 10.1534/genetics.115.183814 26715666 PMC4788249

[mec14560-bib-0044] Mallet, J. , & Barton, N. H. (1989). Strong natural selection in a warning‐color hybrid zone. Evolution, 43, 421–431. 10.1111/j.1558-5646.1989.tb04237.x 28568556

[mec14560-bib-0045] Mallet, J. , Jiggins, C. D. , & McMillan, O. W. (1998). Mimicry and warning colour at the boundary between races and species In Howard D. J., & Berlocher S. H. (Eds.), Endless forms. Species and speciation (pp. 390–403). New York: Oxford University Press.

[mec14560-bib-0046] Mallet, J. , McMillan, W. O. , & Jiggins, C. D. (1998). Mimicry and warning color at the boundary between microevolution and macroevolution In Howard D. & Berlocher S. (Eds.), Endless forms: Species and speciation (pp. 390–403). New York: Oxford University Press.

[mec14560-bib-0047] Mantel, N. (1967). The detection of disease clustering and a generalized regression approach. Cancer Research, 27, 209–220. 6018555

[mec14560-bib-0048] Martin, S. H. , Dasmahapatra, K. K. , Nadeau, N. J. , Salazar, C. , Walters, J. R. , Simpson, F. , … Jiggins, C. D. (2013). Genome‐wide evidence for speciation with gene flow in *Heliconius* butterflies. Genome Research, 23, 1817–1828. 10.1101/gr.159426.113 24045163 PMC3814882

[mec14560-bib-0049] Martin, S. H. , Möst, M. , Palmer, W. J. , Salazar, C. , McMillan, W. O. , Jiggins, F. M. , & Jiggins, C. D. (2016). Natural selection and genetic diversity in the butterfly *Heliconius melpomene* . Genetics, 203, 525–541. 10.1534/genetics.115.183285 27017626 PMC4858797

[mec14560-bib-0050] McMillan, W. O. , Jiggins, C. D. , & Mallet, J. (1997). What initiates speciation in passion‐vine butterflies? Proceedings of the National Academy of Sciences of the United States of America, 94, 8628–8633. 10.1073/pnas.94.16.8628 9238028 PMC23051

[mec14560-bib-0051] Meisel, R. P. , & Connallon, T. (2013). The faster‐X effect: Integrating theory and data. Trends in Genetics, 29, 537–544. 10.1016/j.tig.2013.05.009 23790324 PMC3755111

[mec14560-bib-0052] Mérot, C. , Salazar, C. , Merrill, R. M. , Jiggins, C. , & Joron, M. (2017). What shapes the continuum of reproductive isolation? Lessons from *Heliconius* butterflies. Proceedings of the Royal Society B: Biological Sciences, 284, 20170335 10.1098/rspb.2017.0335 28592669 PMC5474069

[mec14560-bib-0053] Merrill, R. M. , Chia, A. , & Nadeau, N. J. (2014). Divergent warning patterns contribute to assortative mating between incipient *Heliconius* species. Ecology and Evolution, 4, 911–917. 10.1002/ece3.996 24772270 PMC3997309

[mec14560-bib-0054] Merrill, R. M. , Wallbank, R. W. R. , Bull, V. , Salazar, P. C. , Mallet, J. , Stevens, M. , & Jiggins, C. D. (2012). Disruptive ecological selection on a mating cue. Proceedings Biological Sciences/The Royal Society, 279, 4907–4913. 10.1098/rspb.2012.1968 PMC3497240 23075843

[mec14560-bib-0055] Muller, H. J. (1942). Isolating mechanisms, evolution and temperature. Biological Symposia, 6, 71–125.

[mec14560-bib-0056] Muñoz, A. G. , Salazar, C. , Castaño, J. , Jiggins, C. D. , & Linares, M. (2010). Multiple sources of reproductive isolation in a bimodal butterfly hybrid zone. Journal of Evolutionary Biology, 23, 1312–1320. 20456567 10.1111/j.1420-9101.2010.02001.x

[mec14560-bib-0057] Nadachowska‐Brzyska, K. , Burri, R. , Olason, P. I. , Kawakami, T. , Smeds, L. , & Ellegren, H. (2013). Demographic divergence history of pied flycatcher and collared flycatcher inferred from whole‐genome re‐sequencing data. PLoS Genetics, 9, e1003942 10.1371/journal.pgen.1003942 24244198 PMC3820794

[mec14560-bib-0058] Nadeau, N. J. , Martin, S. H. , Kozak, K. M. , Salazar, C. , Dasmahapatra, K. K. , Davey, J. W. , … Jiggins, C. D. (2013). Genome‐wide patterns of divergence and gene flow across a butterfly radiation. Molecular Ecology, 22, 814–826. 10.1111/j.1365-294X.2012.05730.x 22924870

[mec14560-bib-0059] Nadeau, N. J. , Whibley, A. , Jones, R. T. , Davey, J. W. , Dasmahapatra, K. K. , Baxter, S. W. , … Jiggins, C. D. (2012). Genomic islands of divergence in hybridizing *Heliconius* butterflies identified by large‐scale targeted sequencing. Philosophical Transactions of the Royal Society of London Series B, Biological Sciences, 367, 343–353. 10.1098/rstb.2011.0198 22201164 PMC3233711

[mec14560-bib-0060] Naisbit, R. E. , Jiggins, C. D. , Linares, M. , Salazar, C. , & Mallet, J. (2002). Hybrid sterility, Haldane's Rule and speciation in Heliconius cydno and H. melpomene. Genetics, 161, 1517–1526. 12196397 10.1093/genetics/161.4.1517 PMC1462209

[mec14560-bib-0061] Naisbit, R. E. , Jiggins, C. D. , & Mallet, J. (2001). Disruptive sexual selection against hybrids contributes to speciation between *Heliconius cydno* and *Heliconius melpomene* . Proceedings of the Royal Society B, 268, 1849–1854. 10.1098/rspb.2001.1753 11522205 PMC1088818

[mec14560-bib-0062] Nei, M. , & Li, W. H. (1979). Mathematical model for studying genetic variation in terms of restriction endonucleases. Proceedings of the National Academy of Sciences of the United States of America, 76, 5269–5273. 10.1073/pnas.76.10.5269 291943 PMC413122

[mec14560-bib-0063] Oksanen, J. , Blanchet, F. G. , Kindt, R. , Legendre, P. , O'hara, R. B. , Simpson, G. L. , … Wagner, H. (2016). vegan: Community ecology package. R package. Vienna, Austria: R Foundation for Statistical Computing.

[mec14560-bib-0064] Orr, H. A. (1996). Dobzhansky, Bateson, and the genetics of speciation. Genetics, 144, 1331–1335. 8978022 10.1093/genetics/144.4.1331 PMC1207686

[mec14560-bib-0065] Patterson, N. , Richter, D. J. , Gnerre, S. , Lander, E. S. , & Reich, D. (2006). Genetic evidence for complex speciation of humans and chimpanzees. Nature, 441, 1103–1108. 10.1038/nature04789 16710306

[mec14560-bib-0066] Poelstra, J. W. , Vijay, N. , Bossu, C. M. , Lantz, H. , Ryll, B. , Müller, I. , … Wolf, J. B. (2014). The genomic landscape underlying phenotypic integrity in the face of gene flow in crows. Science, 344, 1410–1414. 10.1126/science.1253226 24948738

[mec14560-bib-0067] Pool, J. E. , & Nielsen, R. (2007). Population size changes reshape genomic patterns of diversity. Evolution, 29, 997–1003. 10.1111/j.1558-5646.2007.00238.x PMC3443680 17971168

[mec14560-bib-0068] Presgraves, D. C. (2002). Patterns of postzygotic isolation in Lepidoptera. Evolution, 56, 1168–1183. 10.1111/j.0014-3820.2002.tb01430.x 12144018

[mec14560-bib-0069] Presgraves, D. C. (2008). Sex chromosomes and speciation in *Drosophila* . Trends in Genetics, 24, 336–343. 10.1016/j.tig.2008.04.007 18514967 PMC2819171

[mec14560-bib-0070] Price, A. L. , Patterson, N. J. , Plenge, R. M. , Weinblatt, M. E. , Shadick, N. A. , & Reich, D. (2006). Principal components analysis corrects for stratification in genome‐wide association studies. Nature Genetics, 38, 904–909. 10.1038/ng1847 16862161

[mec14560-bib-0071] Prowell, D. (1998). Sex linkage and speciation in Lepidoptera In Howard D., & Berlocher S. (Eds.), Endless forms. Species and speciation (pp. 309–319). New York: Oxford University Press.

[mec14560-bib-0072] Rambaut, A. , & Grass, N. C. (1997). Seq‐Gen: An application for the Monte Carlo simulation of DNA sequence evolution along phylogenetic trees. Bioinformatics, 13, 235–238. 10.1093/bioinformatics/13.3.235 9183526

[mec14560-bib-0073] Randler, C. (2007). Assortative mating of Carrion *Corvus corone* and Hooded Crows *C. cornix* in the hybrid zone in eastern Germany. Ardea, 95, 143–149. 10.5253/078.095.0116

[mec14560-bib-0074] Sackton, T. B. , Corbett‐Detig, R. B. , Nagaraju, J. , Vaishna, L. , Arunkumar, K. P. , & Hartl, D. L. (2014). Positive selection drives faster‐Z evolution in silkmoths. Evolution, 68, 2331–2342. 24826901 10.1111/evo.12449 PMC4122604

[mec14560-bib-0075] Saether, S. A. , Saetre, G.‐P. , Borge, T. , Wiley, C. , Svedin, N. , Andersson, G. , … Král, M. (2007). Sex chromosome‐linked species recognition and evolution of reproductive isolation in flycatchers. Science, 318, 95–97. 10.1126/science.1141506 17916732

[mec14560-bib-0076] Salazar, C. A. , Jiggins, C. D. , Arias, C. F. , Tobler, A. , Bermingham, E. , & Linares, M. (2005). Hybrid incompatibility is consistent with a hybrid origin of *Heliconius heurippa* Hewitson from its close relatives, *Heliconius cydno* Doubleday and *Heliconius melpomene* Linnaeus. Journal of Evolutionary Biology, 18, 247–256. 15715831 10.1111/j.1420-9101.2004.00839.x

[mec14560-bib-0077] Sánchez, A. P. , Pardo‐Diaz, C. , Enciso‐Romero, J. , Muñoz, A. , Jiggins, C. D. , Salazar, C. , & Linares, M. (2015). An introgressed wing pattern acts as a mating cue. Evolution, 69, 1619–1629. 10.1111/evo.12679 25930106

[mec14560-bib-0078] Sayres, M. A. W. , & Makova, K. D. (2011). Genome analyses substantiate male mutation bias in many species. BioEssays, 33, 938–945. 10.1002/bies.201100091 22006834 PMC4600401

[mec14560-bib-0079] Schiffels, S. , & Durbin, R. (2014). Inferring human population size and separation history from multiple genome sequences. Nature Genetics, 46, 919–925. 10.1038/ng.3015 24952747 PMC4116295

[mec14560-bib-0080] Slatkin, M. , & Voelm, L. (1991). FST in a hierarchial island model. Genetics, 127, 627–629. 2016058 10.1093/genetics/127.3.627 PMC1204389

[mec14560-bib-0081] Smeds, L. , Warmuth, V. , Bolivar, P. , Uebbing, S. , Burri, R. , Suh, A. , … Moreno, J. (2015). Evolutionary analysis of the female‐specific avian W chromosome. Nature Communications, 6, 7330 10.1038/ncomms8330 PMC4468903 26040272

[mec14560-bib-0082] Sperling, F. A. H. (1994). Sex‐linked genes and species differences in Lepidoptera. Canadian Entomologist, 126, 807–818. 10.4039/Ent126807-3

[mec14560-bib-0083] Suomalainen, E. , Cook, L. M. , & Turner, J. R. G. (1973). Achiasmatic oogenesis in the *Heliconiine* butterflies. Hereditas, 74, 302–304.

[mec14560-bib-0084] Tajima, F. (1989). Statistical method for testing the neutral mutation hypothesis by DNA polymorphism. Genetics, 123, 585–595. 2513255 10.1093/genetics/123.3.585 PMC1203831

[mec14560-bib-0085] Thurman, T. J. , Brodie, E. , Evans, E. , & McMillan, W. O. (2018). Facultative pupal mating in *Heliconius erato*: Implications for mate choice, female preference, and speciation. Ecology and Evolution, 8, 1882–1889. 10.1002/ece3.3624 29435261 PMC5792586

[mec14560-bib-0086] Turelli, M. , & Moyle, L. C. (2007). Asymmetric postmating isolation: Darwin's corollary to Haldane's rule. Genetics, 176, 1059–1088. 17435235 10.1534/genetics.106.065979 PMC1894575

[mec14560-bib-0087] Turelli, M. , & Orr, H. A. (1995). The dominance theory of Haldane's rule. Genetics, 140, 389–402. 7635302 10.1093/genetics/140.1.389 PMC1206564

[mec14560-bib-0088] Turner, J. R. G. , & Sheppard, P. M. (1975). Absence of crossover in female butterflies (*Heliconius*). Heredity, 34, 265–269. 10.1038/hdy.1975.29 1055712

[mec14560-bib-0089] Van Belleghem, S. M. , Rastas, P. , Papanicolaou, A. , Martin, S. H. , Arias, C. F. , Supple, M. A. , … Ruiz, M. (2017). Complex modular architecture around a simple toolkit of wing pattern genes. Nature Ecology & Evolution, 1, 52 10.1038/s41559-016-0052 28523290 PMC5432014

[mec14560-bib-0090] Van der Auwera, G. A. , Carneiro, M. O. , Hartl, C. , Poplin, R. , Del Angel, G. , Levy‐Moonshine, A. , … Banks, E. (2013). From fastQ data to high‐confidence variant calls: The genome analysis toolkit best practices pipeline. Current Protocols in Bioinformatics, UNIT 11.10, 1–33. 10.1002/0471250953.bi1110s43 PMC4243306 25431634

[mec14560-bib-0091] Veen, T. , Borge, T. , Griffith, S. C. , Saetre, G. P. , Bures, S. , Gustafsson, L. , & Sheldon, B. C. (2001). Hybridization and adaptive mate choice in flycatchers. Nature, 411, 45–50. 10.1038/35075000 11333971

[mec14560-bib-0092] Vicoso, B. , & Charlesworth, B. (2006). Evolution on the X chromosome: Unusual patterns and processes. Nature Reviews. Genetics, 7, 645–653. 10.1038/nrg1914 16847464

[mec14560-bib-0093] Vicoso, B. , & Charlesworth, B. (2009). Recombination rates may affect the ratio of X to autosomal noncoding polymorphism in African populations of *Drosophila melanogaster* . Genetics, 181, 1699–1701. 10.1534/genetics.108.098004 19189953 PMC2666533

[mec14560-bib-0094] Vijay, N. , Bossu, C. M. , Poelstra, J. W. , Weissensteiner, M. H. , Suh, A. , Kryukov, A. P. , & Wolf, J. B. (2016). Evolution of heterogeneous genome differentiation across multiple contact zones in a crow species complex. Nature Communications, 7, 13195 10.1038/ncomms13195 PMC5095515 27796282

[mec14560-bib-0095] Walters, J. R. , Stafford, C. , Hardcastle, T. J. , & Jiggins, C. D. (2012). Evaluating female remating rates in light of spermatophore degradation in *Heliconius* butterflies: Pupal‐mating monandry versus adult‐mating polyandry. Ecological Entomology, 37, 257–268. 10.1111/j.1365-2311.2012.01360.x

[mec14560-bib-0096] Wolf, J. B. W. , & Ellegren, H. (2017). Making sense of genomic islands of differentiation in light of speciation. Nature Reviews Genetics, 18, 87–100. 10.1038/nrg.2016.133 27840429

[mec14560-bib-0097] Wright, S. (1931). Evolution in mendelian populations. Genetics, 16, 97–159. 17246615 10.1093/genetics/16.2.97 PMC1201091

[mec14560-bib-0098] Wu, C.‐I. , & Davis, A. W. (1993). Evolution of postmating reproductive isolation: The composite nature of Haldane's rule and its genetic bases. American Naturalist, 142, 187–212. 10.1086/285534 19425975

