# Supplementary material for: Patterns of Z chromosome divergence among Heliconius species highlight the importance of historical demography
Source: Mol Ecol. 2018 Apr 14;27(19):3852–72. doi: 10.1111/mec.14560 (PMC6151167; doi:10.1111/mec.14560)
Supplement: Supplementary file 1 [file MEC-27-3852-s001.pdf]

MOLECULAR ECOLOGY

Supplemental Information for:

Patterns of Z chromosome divergence among *Heliconius* species highlight the importance of historical demography

Steven M. Van Belleghem[1,2,3,4], Margarita Baquero[2], Riccardo Papa[3], Camilo Salazar[5], W. Owen McMillan[4], Brian A Counterman[2], Chris D. Jiggins[1] and Simon H. Martin[1]

[1.] Department of Zoology, University of Cambridge, Cambridge CB2 3EJ, United Kingdom.

[2.] Department of Biological Sciences, Mississippi State University, 295 Lee Boulevard, Mississippi State, MS 39762, USA.

[3.] Department of Biology, Center for Applied Tropical Ecology and Conservation, University of Puerto Rico, Rio Piedras, Puerto Rico.

[4.] Smithsonian Tropical Research Institute, Apartado 0843-03092, Panamá, Panama.

[5.] Biology Program, Faculty of Natural Sciences and Mathematics, Universidad del Rosario, Carrera. 24 No. 63C-69, Bogota, D.C. 111221, Colombia.

Corresponding author: vanbelleghemsteven@hotmail.com

MOLECULAR ECOLOGY

Table of Contents:

Table S 1. *H. erato* clade samples. 3
Table S 2. *H. melpomene* clade samples. 6
Table S 3. *H. erato* clade population localities. 9
Table S 4. *H. melpomene* clade population localities. 10
Table S 5. Bash pseudocode to run msms (Ewing & Hermisson 2010) simulations. 11

Figure S 1. *Heliconius erato* and *Heliconius melpomene* clade sampling (A. and B.) and PCA plots of autosomal SNP variation including *H. m. nanna* (C.). 12
Figure S 2. Inference of historical effective population size changes from *H. erato* clade samples using Pairwise Sequentially Markovian Coalescent (PSMC') analysis. 13
Figure S 3. Inference of historical effective population size changes from *H. melpomene* clade samples using Pairwise Sequentially Markovian Coalescent (PSMC') analysis. 14
Figure S 4. F_{ST} plots of hybridizing *H. erato* and *H. melpomene* clade populations. 15
Figure S 5. d_{XY} plots of hybridizing *H. erato* and *H. melpomene* clade populations. 16
Figure S 6. d_a plots of hybridizing *H. erato* and *H. melpomene* clade populations. 17

MOLECULAR ECOLOGY

Table S 1. *H. erato* clade samples. Sex was determined from sequencing heterozygosity on the Z chromosome. SequenceIDs with * are new to this study.

SequenceID	EarthCapeID	Taxon name	Country	Sex	Longitude	*Latitude*	Accession
BC2115	BC2115	*Heliconius erato amalfreda*	Suriname	m	-4.946897	*55.183386*	SAMN05224103
BC2124	BC2124	*Heliconius erato amalfreda*	Suriname	m	-5.943486	*55.186072*	SAMN05224104
STRI_WOM_5779	STRI_WOM_5779	*Heliconius erato amalfreda*	Suriname	m	-5.940653	*55.189922*	SAMN05224208
STRI_WOM_5780	STRI_WOM_5780	*Heliconius erato amalfreda*	Suriname	f	-5.940653	*55.189922*	SAMN05224209
STRI_WOM_5781	STRI_WOM_5781	*Heliconius erato amalfreda*	Suriname	f	-4.932733	*55.200803*	SAMN05224210
STRI_WOM_0057	STRI_WOM_0057	*Heliconius erato chestertonii*	Colombia	m	3.884017	*-76.589367*	SAMN05224192
STRI_WOM_0058	STRI_WOM_0058	*Heliconius erato chestertonii*	Colombia	f	3.884017	*-76.589367*	SAMN05224193
STRI_WOM_0059	STRI_WOM_0059	*Heliconius erato chestertonii*	Colombia	m	3.884017	*-76.589367*	SAMN05224194
3661	CS003661	*Heliconius erato chestertonii*	Colombia	m	3.884017	*-76.589367*	SAMN05224096
3662	CS003662	*Heliconius erato chestertonii*	Colombia	m	3.884017	*-76.589367*	SAMN05224097
3663	CS003663	*Heliconius erato chestertonii*	Colombia	m	3.884017	*-76.589367*	SAMN05224098
3664	CS003664	*Heliconius erato chestertonii*	Colombia	m	3.884017	*-76.589367*	SAMN05224099
cyrbia_004	CYR004	*Heliconius erato cyrbia*	Ecuador	f	-3.726389	*-79.836667*	SAMN05224122
cyrbia_005	CYR005	*Heliconius erato cyrbia*	Ecuador	f	-3.726389	*-79.836667*	SAMN05224123
cyrbia_023	CYR023	*Heliconius erato cyrbia*	Ecuador	m	-3.726389	*-79.836667*	SAMN05224124
cyrbia_024	CYR024	*Heliconius erato cyrbia*	Ecuador	m	-3.726389	*-79.836667*	SAMN05224125
Pet_ED3	Pet_ED3	*Heliconius erato demophoon*	Panama	m	-9.129444	*79.715278*	SAMN05224182
Pet_ED4	Pet_ED4	*Heliconius erato demophoon*	Panama	m	-9.129444	*79.715278*	SAMN05224183
Pet_ED5	Pet_ED5	*Heliconius erato demophoon*	Panama	m	-9.129444	*79.715278*	SAMN05224184
Pet_ED6	Pet_ED6	*Heliconius erato demophoon*	Panama	m	-9.129444	*79.715278*	SAMN05224185
STRI_WOM_0033	STRI_WOM_0033	*Heliconius erato demophoon*	Panama	f	-9.152500	*78.689722*	SAMN05224188
STRI_WOM_0082	STRI_WOM_0082	*Heliconius erato demophoon*	Panama	f	-9.152500	*78.689722*	SAMN05224195
STRI_WOM_0087	STRI_WOM_0087	*Heliconius erato demophoon*	Panama	m	-9.152500	*78.689722*	SAMN05224196
STRIWOM1284	STRI_WOM_1284	*Heliconius erato demophoon*	Panama	m	-9.152500	*78.689722*	SAMN05224198
STRIWOM5353	STRI_WOM_5353	*Heliconius erato demophoon*	Panama	f	-9.152500	*78.689722*	SAMN05224202
STRIWOM5362	STRI_WOM_5362	*Heliconius erato demophoon*	Panama	f	-9.152500	*78.689722*	SAMN05224203
BC2563*	BC_2563	*Heliconius erato emma*	Peru	f	-5.294990	*-78.381000*	SAMN08049955
BC2577*	BC_2577	*Heliconius erato emma*	Peru	m	-5.294990	*-78.381000*	SAMN08049956
BC2578*	BC_2578	*Heliconius erato emma*	Peru	m	-5.294990	*-78.381000*	SAMN08049957
BC2579*	BC_2579	*Heliconius erato emma*	Peru	m	-5.294990	*-78.381000*	SAMN08049958
GS020redo	GS020	*Heliconius erato emma*	Peru	m	-6.181944	*-76.247222*	SAMN05224127
GS021redo	GS021	*Heliconius erato emma*	Peru	f	-6.181944	*-76.247222*	SAMN05224128
NCS_1671	NCS1671	*Heliconius erato emma*	Peru	m	-6.181944	*-76.247222*	SAMN05224154
NCS_1672	NCS1672	*Heliconius erato emma*	Peru	m	-6.181944	*-76.247222*	SAMN05224155
NCS_1673	NCS1673	*Heliconius erato emma*	Peru	m	-6.181944	*-76.247222*	SAMN05224156
NCS_1674	NCS1674	*Heliconius erato emma*	Peru	m	-6.181944	*-76.247222*	SAMN05224157

MOLECULAR ECOLOGY

SequenceID	EarthCapeID	Taxon name	Country	Sex	Longitude	*Latitude*	Accession
NCS_1675	NCS1675	*Heliconius erato emma*	Peru	m	-6.181944	*-76.247222*	SAMN05224158
NCS_2005	NCS2005	*Heliconius erato erato*	French Guiana	m	-4.638611	*52.301667*	SAMN05224160
NCS_2012	NCS2012	*Heliconius erato erato*	French Guiana	f	-4.638611	*52.301667*	SAMN05224161
NCS_2020	NCS2020	*Heliconius erato erato*	French Guiana	m	-4.585000	*52.245556*	SAMN05224162
NCS_2023	NCS2023	*Heliconius erato erato*	French Guiana	m	-4.638611	*52.301667*	SAMN05224163
NCS_2025	NCS2025	*Heliconius erato erato*	French Guiana	m	-4.585000	*52.245556*	SAMN05224164
NCS_2556	NCS2556	*Heliconius erato erato*	French Guiana	m	-4.621944	*52.376111*	SAMN05224174
BC_3277	BC_3277	*Heliconius erato etylus*	Ecuador	m	-1.977860	*-78.009450*	SAMN05224110
BC_3278	BC_3278	*Heliconius erato etylus*	Ecuador	f	-1.977860	*-78.009450*	SAMN05224111
BC_3280	BC_3280	*Heliconius erato etylus*	Ecuador	f	-1.977860	*-78.009450*	SAMN05224112
BC_3281	BC_3281	*Heliconius erato etylus*	Ecuador	m	-1.977860	*-78.009450*	SAMN05224113
BC_3282	BC_3282	*Heliconius erato etylus*	Ecuador	f	-1.977860	*-78.009450*	SAMN05224114
BC2635*	BC_2635	*Heliconius erato favorinus*	Peru	f	-6.417400	*-77.443290*	SAMN08049959
BC2637*	BC_2637	*Heliconius erato favorinus*	Peru	m	-6.417400	*-77.443290*	SAMN08049960
BC2638*	BC_2638	*Heliconius erato favorinus*	Peru	m	-6.417400	*-77.443290*	SAMN08049961
BC2639*	BC_2639	*Heliconius erato favorinus*	Peru	m	-6.417400	*-77.443290*	SAMN08049962
GS012redo	GS012	*Heliconius erato favorinus*	Peru	m	-6.461389	*-76.341944*	SAMN05224126
NCS_0471	NCS0471	*Heliconius erato favorinus*	Peru	m	-6.474167	*-76.010278*	SAMN05224148
NCS_0473	NCS0473	*Heliconius erato favorinus*	Peru	m	-6.474167	*-76.010278*	SAMN05224149
NCS_0476	NCS0476	*Heliconius erato favorinus*	Peru	m	-6.474167	*-76.010278*	SAMN05224150
NCS_0478	NCS0478	*Heliconius erato favorinus*	Peru	f	-6.474167	*-76.010278*	SAMN05224151
NCS_0479	NCS0479	*Heliconius erato favorinus*	Peru	m	-6.474167	*-76.010278*	SAMN05224152
NCS_2554	NCS2554	*Heliconius erato favorinus*	Peru	f	-6.474167	*-76.010278*	SAMN05224172
NCS_2555	NCS2555	*Heliconius erato favorinus*	Peru	m	-6.474167	*-76.010278*	SAMN05224173
STRI_WOM_0042	STRI_WOM_0042	*Heliconius erato hydara*	Panama	f	-9.152500	*78.689722*	SAMN05224191
NCS_1179	NCS1179	*Heliconius erato hydara*	French Guiana	m	-4.703611	*52.303611*	SAMN05224153
NCS_1979	NCS1979	*Heliconius erato hydara*	French Guiana	m	-4.571667	*52.223333*	SAMN05224159
NCS_2080	NCS2080	*Heliconius erato hydara*	French Guiana	f	-4.607778	*52.272500*	SAMN05224165
NCS_2211	NCS2211	*Heliconius erato hydara*	French Guiana	m	-4.547222	*52.170278*	SAMN05224166
NCS_2217	NCS2217	*Heliconius erato hydara*	French Guiana	m	-4.544444	*52.152500*	SAMN05224167
STRI_WOM_0039	STRI_WOM_0039	*Heliconius erato hydara*	Panama	m	-9.152500	*78.689722*	SAMN05224189
STRI_WOM_0040	STRI_WOM_0040	*Heliconius erato hydara*	Panama	m	-9.152500	*78.689722*	SAMN05224190
STRI_WOM_0088	STRI_WOM_0088	*Heliconius erato hydara*	Panama	m	-9.152500	*78.689722*	SAMN05224197
STRI_WOM_5193	STRI_WOM_5193	*Heliconius erato hydara*	Panama	m	-9.152500	*78.689722*	SAMN05224200
STRI_WOM_5351	STRI_WOM_5351	*Heliconius erato hydara*	Panama	m	-9.152500	*78.689722*	SAMN05224201
BC_0411	BC0411	*Heliconius erato lativitta*	Ecuador	m	-1.098333	*77.583889*	SAMN05224101
lativitta_01	LAT01	*Heliconius erato lativitta*	Ecuador	f	-1.098333	*77.583889*	SAMN05224137
lativitta_02	LAT02	*Heliconius erato lativitta*	Ecuador	f	-1.098333	*77.583889*	SAMN05224138
lativitta_03	LAT03	*Heliconius erato lativitta*	Ecuador	f	-1.098333	*77.583889*	SAMN05224139

MOLECULAR ECOLOGY

SequenceID	EarthCapeID	Taxon name	Country	Sex	Longitude	*Latitude*	Accession
lativitta_04	LAT04	*Heliconius erato lativitta*	Ecuador	f	-0.712500	*77.583889*	SAMN05224140
BC_0410	BC_0410	*Heliconius erato notabilis*	Ecuador	m	-1.813370	*-78.045070*	SAMN05224100
BC_3223	BC_3223	*Heliconius erato notabilis*	Ecuador	m	-1.813370	*-78.045070*	SAMN05224105
BC_3224	BC_3224	*Heliconius erato notabilis*	Ecuador	f	-1.813370	*-78.045070*	SAMN05224106
BC_3225	BC_3225	*Heliconius erato notabilis*	Ecuador	f	-1.813370	*-78.045070*	SAMN05224107
BC_3227	BC_3227	*Heliconius erato notabilis*	Ecuador	f	-1.822590	*-78.044060*	SAMN05224108
BC_3228	BC_3228	*Heliconius erato notabilis*	Ecuador	m	-1.822590	*-78.044060*	SAMN05224109
notabilis_01	NOT01	*Heliconius erato notabilis*	Ecuador	m	-1.399167	*78.181111*	SAMN05224178
notabilis_02	NOT02	*Heliconius erato notabilis*	Ecuador	m	-1.399167	*78.181111*	SAMN05224179
notabilis_03	NOT03	*Heliconius erato notabilis*	Ecuador	m	-1.399167	*78.181111*	SAMN05224180
notabilis_04	NOT04	*Heliconius erato notabilis*	Ecuador	m	-1.399167	*78.181111*	SAMN05224181
CA51	CA51	*Heliconius erato petiverana*	Mexico	f	18.957903	*-90.269233*	SAMN05224115
CA53	CA53	*Heliconius erato petiverana*	Mexico	m	18.957903	*-90.269233*	SAMN05224116
CA54	CA54	*Heliconius erato petiverana*	Mexico	m	18.957903	*-90.269233*	SAMN05224117
CA55	CA55	*Heliconius erato petiverana*	Mexico	f	18.957903	*-90.269233*	SAMN05224118
CA56	CA56	*Heliconius erato petiverana*	Mexico	m	18.957903	*-90.269233*	SAMN05224119
STRI_WOM_5732	STRI_WOM_5732	*Heliconius erato phyllis*	Bolivia	m	-18.176869	*-63.881664*	SAMN05224204
STRI_WOM_5742	STRI_WOM_5742	*Heliconius erato phyllis*	Bolivia	m	-18.176869	*-63.881664*	SAMN05224205
STRI_WOM_5765	STRI_WOM_5765	*Heliconius erato phyllis*	Bolivia	m	-18.176869	*-63.881664*	SAMN05224206
STRI_WOM_5766	STRI_WOM_5766	*Heliconius erato phyllis*	Bolivia	m	-18.176869	*-63.881664*	SAMN05224207
M_3654	CS003654	*Heliconius erato venus*	Colombia	m	3.531100	*-76.753383*	SAMN05224141
M_3655	CS003655	*Heliconius erato venus*	Colombia	f	3.531100	*-76.753383*	SAMN05224142
M_3656	CS003656	*Heliconius erato venus*	Colombia	m	3.531100	*-76.753383*	SAMN05224143
M_3657	CS003657	*Heliconius erato venus*	Colombia	m	3.531100	*-76.753383*	SAMN05224144
M_3659	CS003659	*Heliconius erato venus*	Colombia	m	3.531100	*-76.753383*	SAMN05224145
BC2565*	BC_2565	*Heliconius himera*	Peru	f	-5.437240	*-78.471400*	SAMN08049963
BC2566*	BC_2566	*Heliconius himera*	Peru	m	-5.437240	*-78.471400*	SAMN08049964
BC2567*	BC_2567	*Heliconius himera*	Peru	m	-5.437240	*-78.471400*	SAMN08049965
BC2570*	BC_2570	*Heliconius himera*	Peru	f	-5.437240	*-78.471400*	SAMN08049966
himera_001	HIM001	*Heliconius himera*	Ecuador	m	-4.276111	*-79.195833*	SAMN05224132
himera_002	HIM002	*Heliconius himera*	Ecuador	m	-4.276111	*-79.195833*	SAMN05224133
himera_003	HIM003	*Heliconius himera*	Ecuador	f	-4.276111	*-79.195833*	SAMN05224134
himera_006	HIM006	*Heliconius himera*	Ecuador	f	-4.276111	*-79.195833*	SAMN05224135
himera_030	HIM030	*Heliconius himera*	Ecuador	m	-4.276111	*-79.195833*	SAMN05224136

MOLECULAR ECOLOGY

Table S 2. *H. melpomene* clade samples. Sex was determined from sequencing heterozygosity on the Z chromosome. SequenceIDs with * are new to this study. SequenceIDs with † were obtained from stocks.

SequenceID	EarthCapeID	Taxon name	Country	Sex	Longitude	Latitude	Accession
agl.JM108	JM-09-108	*Heliconius melpomene aglaope*	Peru	m	-5.910300	-76.225800	SAMEA1919251
agl.JM112	JM-09-112	*Heliconius melpomene aglaope*	Peru	m	-5.910300	-76.225800	SAMEA1919264
agl.JM569	JM-11-569	*Heliconius melpomene aglaope*	Peru	m	-5.945800	-76.245300	SAMEA1919274
agl.JM572	JM-11-572	*Heliconius melpomene aglaope*	Peru	m	-5.945800	-76.246600	SAMEA1919259
ama.JM160	JM-11-160	*Heliconius melpomene amaryllis*	Peru	f	-5.675600	-77.674700	SAMEA1919261
ama.JM216	JM-09-216	*Heliconius melpomene amaryllis*	Peru	m	-6.468500	-76.353300	SAMEA1919261
ama.JM293	JM-11-293	*Heliconius melpomene amaryllis*	Peru	f	-6.470300	-76.347300	SAMEA1919277
ama.JM48	JM-11-48	*Heliconius melpomene amaryllis*	Peru	m	-6.096000	-76.977400	SAMEA1919269
ama.MJ11-3188*	MJ11-3188	*Heliconius melpomene amaryllis*	Peru	m	-5.672840	-77.719470	SAMEA104585061
ama.MJ11-3189*	MJ11-3189	*Heliconius melpomene amaryllis*	Peru	m	-5.672840	-77.719470	SAMEA104585062
ama.MJ11-3202*	MJ11-3202	*Heliconius melpomene amaryllis*	Peru	m	-5.674530	-77.671140	SAMEA104585063
ama.MJ12-3217*	MJ12-3217	*Heliconius melpomene amaryllis*	Peru	m	-6.454740	-76.299440	SAMEA104585064
ama.MJ12-3258*	MJ12-3258	*Heliconius melpomene amaryllis*	Peru	m	-6.452960	-76.287620	SAMEA104585065
ama.MJ12-3301*	MJ12-3301	*Heliconius melpomene amaryllis*	Peru	m	-6.452830	-76.286215	SAMEA104585066
chi.CAM25091*†	CAM025091	*Heliconius cydno chioneus*	Panama	f	9.120000	-79.702000	SAMEA104585050
chi.CAM25137*†	CAM025137	*Heliconius cydno chioneus*	Panama	f	9.120000	-79.702000	SAMEA104585051
chi.CAM580	CAM000580	*Heliconius cydno chioneus*	Panama	m	9.120000	-79.702000	SAMEA104585044
chi.CAM582	CAM000582	*Heliconius cydno chioneus*	Panama	m	9.120000	-79.702000	SAMEA104585045
chi.CAM585*	CAM000585	*Heliconius cydno chioneus*	Panama	m	9.120000	-79.702000	SAMEA104585047
chi.CAM586*	CAM000586	*Heliconius cydno chioneus*	Panama	m	9.120000	-79.702000	SAMEA104585048
chi.CJ553	CAM000553	*Heliconius cydno chioneus*	Panama	m	9.171400	-79.757300	SAMEA1919256
chi.CJ560	CAM000560	*Heliconius cydno chioneus*	Panama	m	9.171400	-79.757300	SAMEA1919265
chi.CJ564	CAM000564	*Heliconius cydno chioneus*	Panama	m	9.171400	-79.757300	SAMEA1919278
chi.CJ565	CAM000565	*Heliconius cydno chioneus*	Panama	m	9.171400	-79.757300	SAMEA1919262
cor.CS3*	CS002167	*Heliconius cydno cordula*	Venezuela	m	7.798888	-72.198888	SAMEA104585052
cor.CS4*	CS002258	*Heliconius cydno cordula*	Venezuela	m	7.798888	-72.198888	SAMEA104585053
cor.STRI7	STRI007	*Heliconius cydno cordula*	Venezuela	m	7.798888	-72.198888	SAMEA3670511
flo.CS12*	CS002395	*Heliconius timareta florencia*	Colombia	m	1.709650	-75.697583	SAMEA104585100
flo.CS13*	CS002402	*Heliconius timareta florencia*	Colombia	m	1.709650	-75.697583	SAMEA104585101
flo.CS14*	CS002403	*Heliconius timareta florencia*	Colombia	m	1.709650	-75.697583	SAMEA104585102
flo.CS15*	CS002406	*Heliconius timareta florencia*	Colombia	m	1.709650	-75.697583	SAMEA104585103
flo.CS2337*	CS002337	*Heliconius timareta florencia*	Colombia	m	1.710833	-75.708889	SAMEA104585104
flo.CS2338*	CS002338	*Heliconius timareta florencia*	Colombia	m	1.710833	-75.708889	SAMEA104585105
flo.CS2341*	CS002341	*Heliconius timareta florencia*	Colombia	m	1.813611	-75.668611	SAMEA104585106
flo.CS2350*	CS002350	*Heliconius timareta florencia*	Colombia	m	1.710833	-75.708889	SAMEA104585107
flo.CS2358*	CS002358	*Heliconius timareta florencia*	Colombia	m	1.710833	-75.708889	SAMEA104585108
flo.CS2359*	CS002359	*Heliconius timareta florencia*	Colombia	m	1.710833	-75.708889	SAMEA104585109

MOLECULAR ECOLOGY

SequenceID	EarthCapeID	Taxon name	Country	Sex	Longitude	Latitude	Accession
heu.CS20*	CS00CH21	*Heliconius heurippa*	Colombia	f	4.175000	-73.678056	SAMEA104585060
heu.CS9	CS002020	*Heliconius heurippa*	Colombia	m	4.175000	-73.678056	SAMEA1322902
heu.STRI2	STRI_002	*Heliconius heurippa*	Colombia	f	4.175000	-73.678056	SAMEA3670535
mal.CS1002*	CS001002	*Heliconius melpomene malleti*	Colombia	m	1.803333	-75.655277	SAMEA104585067
mal.CS1011*	CS001011	*Heliconius melpomene malleti*	Colombia	m	1.803333	-75.655277	SAMEA104585068
mal.CS1815*	CS001815	*Heliconius melpomene malleti*	Colombia	m	1.803333	-75.655277	SAMEA104585069
mal.CS21	CS002311	*Heliconius melpomene malleti*	Colombia	m	*1.813611*	*-75.668611*	SAMEA3723397
mal.CS22	CS001286	*Heliconius melpomene malleti*	Colombia	m	*1.609722*	*-75.666944*	SAMEA3723398
mal.CS24	CS001321	*Heliconius melpomene malleti*	Colombia	m	*1.750556*	*-75.631944*	SAMEA3723399
mal.CS586*	CS000586	*Heliconius melpomene malleti*	Colombia	m	1.803333	-75.655277	SAMEA104585071
mal.CS594*	CS000594	*Heliconius melpomene malleti*	Colombia	f	1.803333	-75.655277	SAMEA104585072
mal.CS604*	CS000604	*Heliconius melpomene malleti*	Colombia	m	1.803333	-75.655277	SAMEA104585073
mal.CS615*	CS000615	*Heliconius melpomene malleti*	Colombia	m	1.803333	-75.655277	SAMEA104585074
melC.CS25	CS000CM4	*Heliconius melpomene melpomene*	Colombia	m	4.924724	-68.925111	SAMEA3723400
melC.CS26	CS000CM5	*Heliconius melpomene melpomene*	Colombia	m	4.924724	-68.925111	SAMEA3723401
melC.CS27	CS00CM10	*Heliconius melpomene melpomene*	Colombia	m	4.924724	-68.925111	SAMEA3723402
melC.CS3	CS000CM3	*Heliconius melpomene melpomene*	Colombia	m	4.924724	-68.925111	SAMEA3723393
melC.CS6	CS000CM6	*Heliconius melpomene melpomene*	Colombia	m	4.924724	-68.925111	SAMEA3723394
melG.CAM1349*	CAM001349	*Heliconius melpomene melpomene*	French Guiana	f	2.522209	-51.193404	SAMEA104585075
melG.CAM1422*	CAM001422	*Heliconius melpomene melpomene*	French Guiana	m	2.522209	-51.193404	SAMEA104585076
melG.CAM2035*	CAM002035	*Heliconius melpomene melpomene*	French Guiana	m	2.522209	-51.193404	SAMEA104585077
melG.CAM8171*	CAM008171	*Heliconius melpomene melpomene*	French Guiana	f	2.522209	-51.193404	SAMEA104585078
melG.CAM8216*	CAM008216	*Heliconius melpomene melpomene*	French Guiana	m	2.522209	-51.193404	SAMEA104585080
melG.CAM8218*	CAM008218	*Heliconius melpomene melpomene*	French Guiana	m	2.522209	-51.193404	SAMEA104585081
melG.CJ13435	CAM013435	*Heliconius melpomene melpomene*	French Guiana	m	2.522209	-51.193404	SAMEA1919276
melG.CJ9315	CAM009315	*Heliconius melpomene melpomene*	French Guiana	m	2.522209	-51.193404	SAMEA1919270
melG.CJ9316	CAM009316	*Heliconius melpomene melpomene*	French Guiana	m	2.522209	-51.193404	SAMEA1919252
melG.CJ9317	CAM009317	*Heliconius melpomene melpomene*	French Guiana	m	2.522209	-51.193404	SAMEA1919267
melP.CJ18038	CAM018038	*Heliconius melpomene melpomene*	Panama	f	8.613600	-78.139800	SAMEA1919255
melP.CJ18097	CAM018097	*Heliconius melpomene melpomene*	Panama	m	8.279700	-77.809800	SAMEA1919258
melP.HGC1†	gen_ref	*Heliconius melpomene melpomene*	Panama	f	*NA*	*NA*	SAMN00794386
melPle.CJ16042	CAM016042	*Heliconius melpomene plesseni*	Ecuador	m	-1.371267	-77.874517	SAMEA3670548
nan.MK14	MK000014	*Heliconius melpomene nanna*	Brazil	m	-12.374009	-48.189253	SAMN04407968
nan.MK62	MK000062	*Heliconius melpomene nanna*	Brazil	m	-12.374009	-48.189253	SAMN04407969
nan.MK63	MK000063	*Heliconius melpomene nanna*	Brazil	m	-12.374009	-48.189253	SAMN04407970
nan.MK64	MK000064	*Heliconius melpomene nanna*	Brazil	m	-12.374009	-48.189253	SAMN04407961
ple.CJ16293	CAM016293	*Heliconius melpomene plesseni*	Ecuador	m	-1.459967	-78.072800	SAMEA3670557
ple.CJ9156	CAM009156	*Heliconius melpomene plesseni*	Ecuador	m	-1.398010	-78.178130	SAMEA3670556
ros.CAM1841	CAM001841	*Heliconius melpomene rosina*	Panama	m	9.076000	-79.659000	SAMEA104585083

MOLECULAR ECOLOGY

SequenceID	EarthCapeID	Taxon name	Country	Sex	Longitude	Latitude	Accession
ros.CAM1880*	CAM001880	*Heliconius melpomene rosina*	Panama	m	9.076000	-79.659000	SAMEA104585084
ros.CAM2045*	CAM002045	*Heliconius melpomene rosina*	Panama	m	9.110300	-79.690700	SAMEA104585085
ros.CAM2059*	CAM002059	*Heliconius melpomene rosina*	Panama	m	9.110300	-79.690700	SAMEA104585086
ros.CAM2519*	CAM002519	*Heliconius melpomene rosina*	Panama	m	9.010900	-79.547700	SAMEA104585087
ros.CAM2552*	CAM002552	*Heliconius melpomene rosina*	Panama	m	9.010900	-79.547700	SAMEA104585088
ros.CJ2071	CAM002071	*Heliconius melpomene rosina*	Panama	m	9.120600	-79.696900	SAMEA1919257
ros.CJ531	CAM000531	*Heliconius melpomene rosina*	Panama	m	9.120600	-79.696900	SAMEA1919271
ros.CJ533	CAM000533	*Heliconius melpomene rosina*	Panama	m	9.120600	-79.696900	SAMEA1919260
ros.CJ546	CAM000546	*Heliconius melpomene rosina*	Panama	m	9.120600	-79.696900	SAMEA1919279
thxn.JM313	JM-09-313	*Heliconius timareta thelxinoe*	Peru	m	-6.458400	-76.287700	SAMEA1919266
thxn.JM57	JM-09-57	*Heliconius timareta thelxinoe*	Peru	m	-6.452800	-76.298700	SAMEA1919254
thxn.JM84	JM-09-84	*Heliconius timareta thelxinoe*	Peru	m	-6.452800	-76.298700	SAMEA1919273
thxn.JM86	JM-09-86	*Heliconius timareta thelxinoe*	Peru	m	-6.452800	-76.298700	SAMEA1919263
thxn.MJ12-3221*	MJ12-3221	*Heliconius timareta thelxinoe*	Peru	m	-5.654640	-77.693750	SAMEA104585110
thxn.MJ12-3233*	MJ12-3233	*Heliconius timareta thelxinoe*	Peru	m	-6.451900	-76.298460	SAMEA104585111
thxn.MJ12-3308*	MJ12-3308	*Heliconius timareta thelxinoe*	Peru	m	-5.654640	-77.693750	SAMEA104585112
txn.MJ11-3339*	MJ11-3339	*Heliconius timareta thelxinoe*	Peru	m	-5.654640	-77.693750	SAMEA104585113
txn.MJ11-3340*	MJ11-3340	*Heliconius timareta thelxinoe*	Peru	m	-5.654640	-77.693750	SAMEA104585114
txn.MJ11-3460*	MJ11-3460	*Heliconius timareta thelxinoe*	Peru	m	-5.654640	-77.693750	SAMEA104585115
vul.CS10	CS000710	*Heliconius melpomene vulcanus*	Colombia	m	*3.900000*	*-76.632500*	SAMEA3723391
vul.CS3603*	CS003603	*Heliconius melpomene vulcanus*	Colombia	m	3.517500	-76.757222	SAMEA104585091
vul.CS3605*	CS003605	*Heliconius melpomene vulcanus*	Colombia	m	3.517500	-76.757222	SAMEA104585092
vul.CS3606*	CS003606	*Heliconius melpomene vulcanus*	Colombia	m	3.517500	-76.757222	SAMEA104585093
vul.CS3612*	CS003612	*Heliconius melpomene vulcanus*	Colombia	m	3.517500	-76.757222	SAMEA104585094
vul.CS3614*	CS003614	*Heliconius melpomene vulcanus*	Colombia	m	3.517500	-76.757222	SAMEA104585095
vul.CS3615*	CS003615	*Heliconius melpomene vulcanus*	Colombia	m	3.517500	-76.757222	SAMEA104585096
vul.CS3617*	CS003617	*Heliconius melpomene vulcanus*	Colombia	m	3.517500	-76.757222	SAMEA104585097
vul.CS3618*	CS003618	*Heliconius melpomene vulcanus*	Colombia	m	3.517500	-76.757222	SAMEA104585098
vul.CS3621*	CS003621	*Heliconius melpomene vulcanus*	Colombia	m	3.517500	-76.757222	SAMEA104585099
zel.CS1	CS002242	*Heliconius cydno zelinde*	Colombia	m	3.939444	-77.368889	SAMEA104106540
zel.CS1028*	CS001028	*Heliconius cydno zelinde*	Colombia	m	3.958333	-77.373333	SAMEA104585054
zel.CS1029*	CS001029	*Heliconius cydno zelinde*	Colombia	m	3.939444	-77.368889	SAMEA104585055
zel.CS1030*	CS001030	*Heliconius cydno zelinde*	Colombia	m	3.939444	-77.368889	SAMEA104585056
zel.CS1033*	CS001033	*Heliconius cydno zelinde*	Colombia	m	3.958333	-77.373333	SAMEA104585057
zel.CS1035*	CS001035	*Heliconius cydno zelinde*	Colombia	m	3.958333	-77.373333	SAMEA104585058
zel.CS2	CS002261	*Heliconius cydno zelinde*	Colombia	m	3.939444	-77.368889	SAMEA104106542
zel.CS2262	CS002262	*Heliconius cydno zelinde*	Colombia	f	3.958333	-77.373333	SAMEA3670517
zel.CS273	CS000273	*Heliconius cydno zelinde*	Colombia	m	3.958333	-77.373333	SAMEA104585059
zel.CS30	CS002260	*Heliconius cydno zelinde*	Colombia	f	3.939444	-77.368889	SAMEA104106543

Table S 3. ***H. erato*** **clade population localities.**

**Population**	**Country**	**Longitude**	**Latitude**
H. e. amalfreda	Suriname	5.340884	-55.190021
H. e. chestertonii	Colombia	3.884017	-76.589367
H. e. cyrbia	Ecuador	-3.726389	-79.836667
H. e. demophoon	Panama	9.129444	-79.715278
H. e. emma	Peru	-6.181944	-76.247222
H. e. emma	Peru	-5.294990	-78.381000
H. e. erato	French Guiana	4.617963	-52.295370
H. e. etylus	Ecuador	-1.977860	-78.009450
H. e. favorinus	Peru	-6.469375	-76.134653
H. e. favorinus	Peru	-6.417400	-77.443290
H. e. hydara	French Guiana	4.594944	-52.224444
H. e. hydara	Panama	9.152500	-78.689722
H. e. lativitta	Ecuador	-1.021167	-77.615222
H. e. notabilis	Ecuador	-1.399167	-78.181111
H. e. notabilis	Ecuador	-1.817058	-78.044666
H. e. petiverana	Mexico	18.957903	-90.269233
H. e. phyllis	Bolivia	-18.176869	-63.881664
H. e. venus	Colombia	3.531100	-76.753383
H. himera	Ecuador	-4.276111	-79.195833
H. himera	Peru	-5.437240	-78.471400

Table S 4. *H. melpomene* clade population localities.

Population	Country	Longitude	Latitude
H. c. chioneus	Panama	9.142844	-79.726578
H. c. cordula	Venezuela	7.798888	-72.198888
H. c. zelinde	Colombia	3.948889	-77.371111
H. heurippa	Colombia	4.175000	-73.678056
H. m. aglaope	Peru	-5.928050	-76.235875
H. m. amaryllis	Peru	-6.109114	-76.933606
H. m. malleti	Colombia	1.779722	-75.655444
H. m. melpomene	Panama	8.446650	-77.974800
H. m. melpomene	Colombia	4.774751	-73.201667
H. m. melpomene	French Guiana	4.928360	-52.416800
H. m. nanna	Brazil	-19.098308	-40.186042
H. m. plesseni	Ecuador	-1.409748	-78.041816
H. m. rosina	Panama	9.087680	-79.658240
H. m. vulcanus	Colombia	3.555750	-76.744750
H. t. florencia	Colombia	1.709650	-75.697583
H. t. thelxinoe	Peru	-6.009900	-77.072479

MOLECULAR ECOLOGY

Table S 5. Bash pseudocode to run *msms* (Ewing & Hermisson 2010) simulations. Note that mutation rate is only used by *seq-gen* when simulating the sequences, population size is used to scale times and migration proportions.

**Population size changes**	for popSize in 1.5e6 2e6 2.25e6 3e6;<br>do for popChange in 128 32 16 8 4 2 1 0.5 0.25 0.125 0.0625 0.03125 0.015625 0.0078125;<br>do for changeTime in<br>do<br><br>changeTimeScaled=$(($(echo ${changeTime}) / (4 * $(echo ${popSize})))<br><br>msms 5 1 -T -I 1 5 -r 500.0 50000 \<br>-en 0 1 $(echo ${popChange}) \<br>-en $(echo ${changeTime}) 1 1;<br><br>done; done; done; done;
**Migration and incompatibiltes**	for popSize in 1.5e6 2e6 2.25e6 3e6;<br>do for splitTime in 1e6 2e6 4e6;<br>do for migration in 1e-6 5e-7 2.5e-7 1.25e-7 6.25e-08 3.125e-08 1.5625e-08;<br>do for migrationReduction in 0 0.2 0.4 0.6 0.8 1;<br>do<br><br>splitTimeScaled=$(($(echo ${splitTime}) / (4 * $(echo ${popSize})))<br>migrationScaled=$((($(echo ${migration}) * (4 * $(echo ${popSize})) \<br>* $(echo ${migrationReduction}))))<br><br>msms 10 1 -T -I 2 5 5 -r 500.0 50000 \<br>-ej $(echo ${splitTimeScaled}) 2 1 \<br>-m 1 2 $(echo ${migrationScaled}) \<br>-m 2 1 $(echo ${migrationScaled});<br><br>done; done; done; done; done;

MOLECULAR ECOLOGY

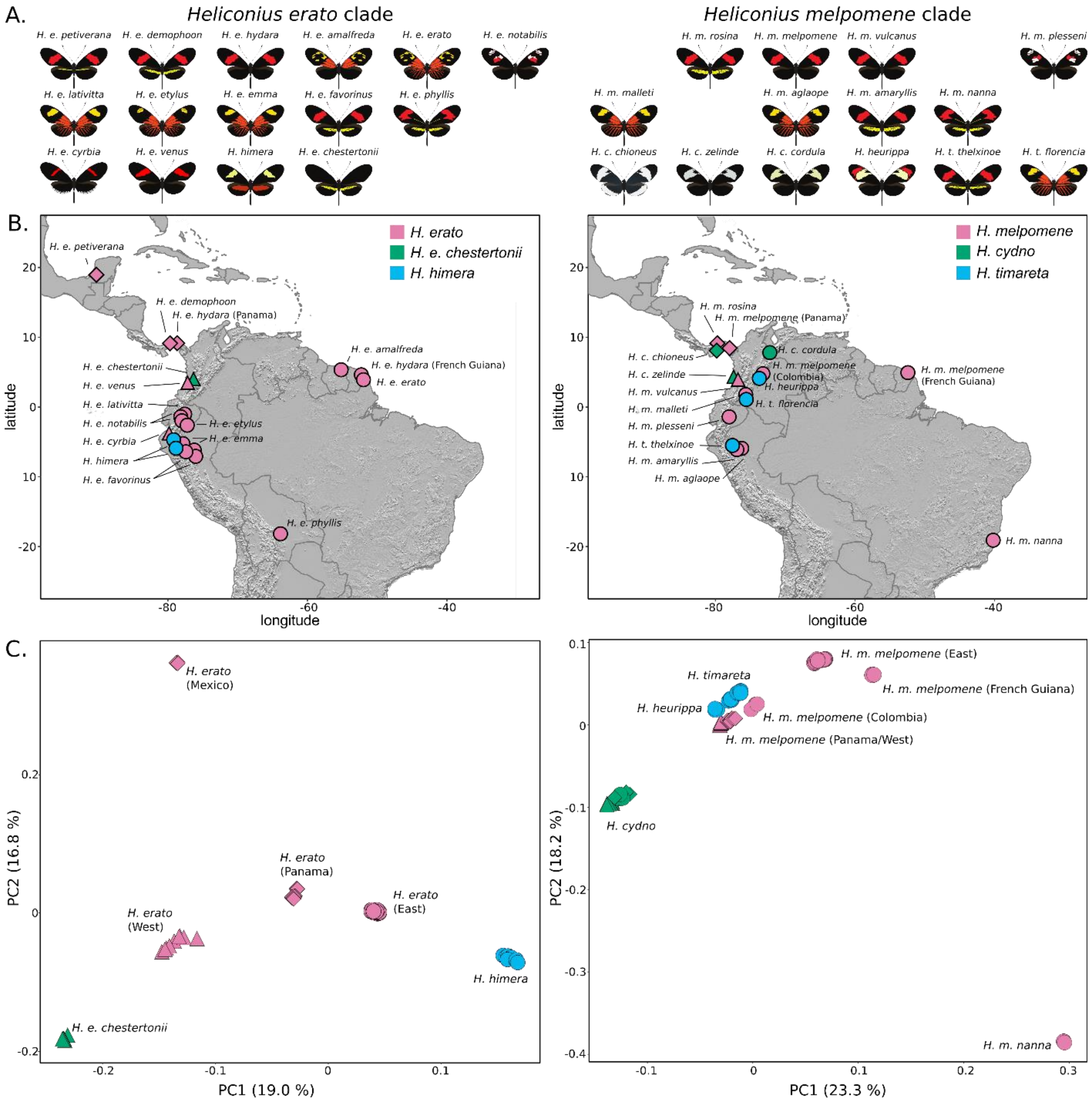

Figure S 1. *Heliconius erato* and *Heliconius melpomene* clade sampling (A. and B.) and PCA plots of autosomal SNP variation including *H. m. nanna* (C.). Colors represent populations that have been identified as separate species from *H. erato* (*H. erato* (pink), *H. e. chestertonii* (green) and *H. himera* (blue)) and *H. melpomene* (*H. melpomene* (pink), *H. timareta* (blue) and *H. cydno* (green)). Shapes represent geographic regions; Mexico and Panama (diamond), West Andes (triangles) and East Andes (circles). Names in (A. and B.) represent race names that have been assigned to populations that display distinct color patterns.

MOLECULAR ECOLOGY

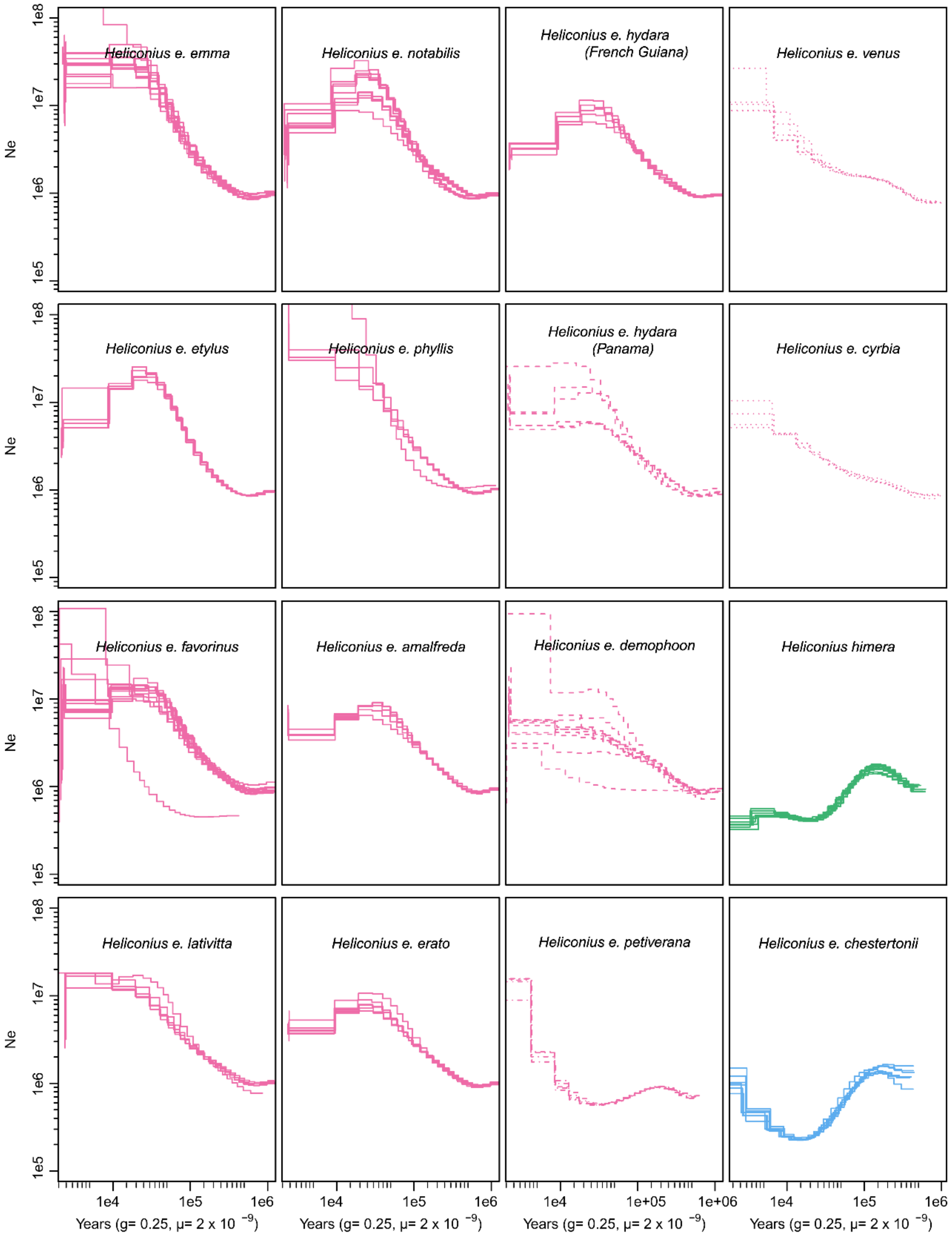

Figure S 2. Inference of historical effective population size changes from *H. erato* clade samples using Pairwise Sequentially Markovian Coalescent (PSMC') analysis. Pink = *H. erato* (full lines = East Andes, dashed lines = Panama, dotted lines = West Andes and dash/dot line s= Mexico), green = *H. himera* and blue = *H. e. chestertonii*.

MOLECULAR ECOLOGY

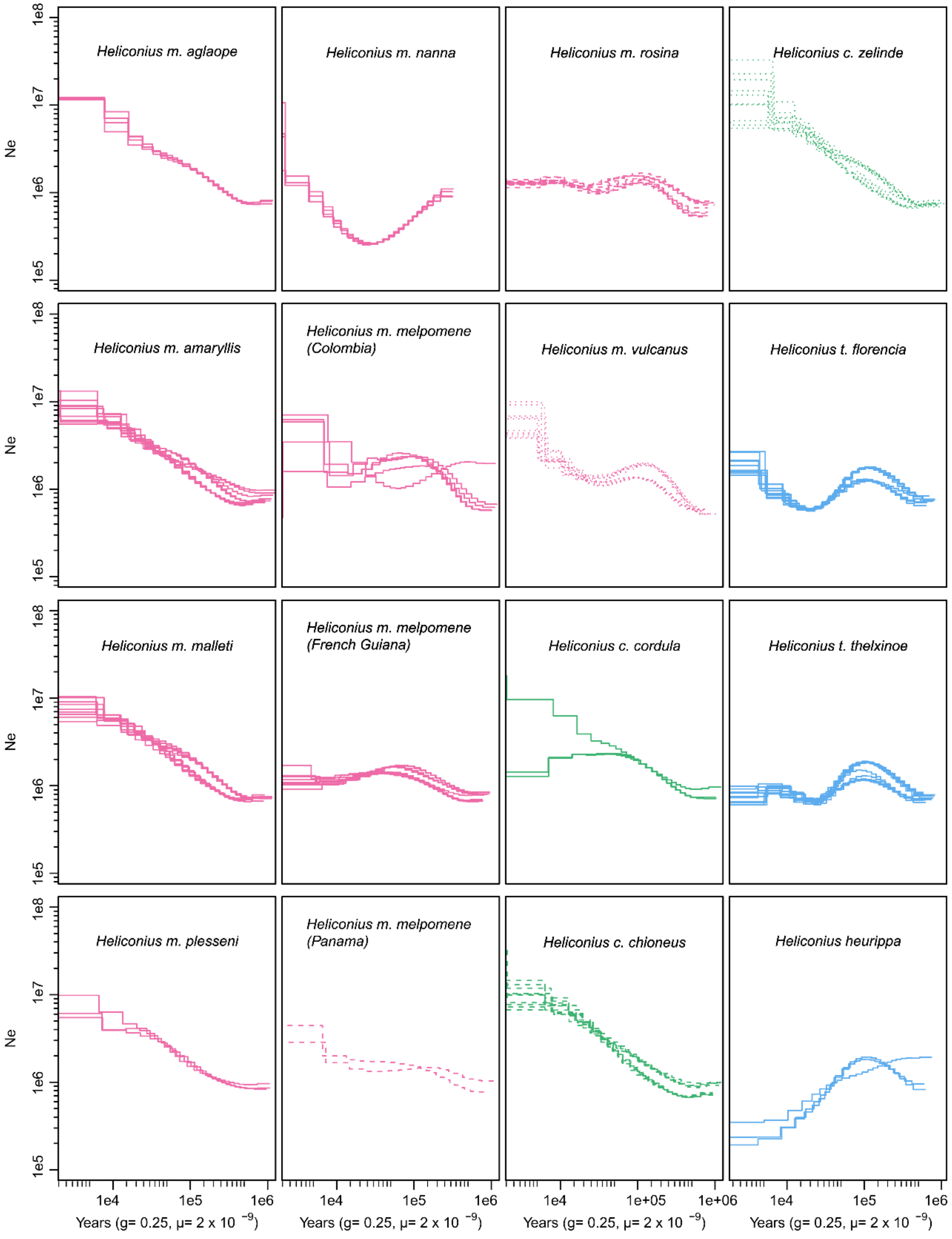

Figure S 3. Inference of historical effective population size changes from *H. melpomene* clade samples using Pairwise Sequentially Markovian Coalescent (PSMC') analysis. Pink = *H. melpomene* (full lines = East Andes, dashed lines = Panama and dotted lines = West Andes), green = *H. cydno* (full lines = East Andes, dashed lines = Panama and dotted lines = West Andes) and blue = *H. timareta*.

MOLECULAR ECOLOGY

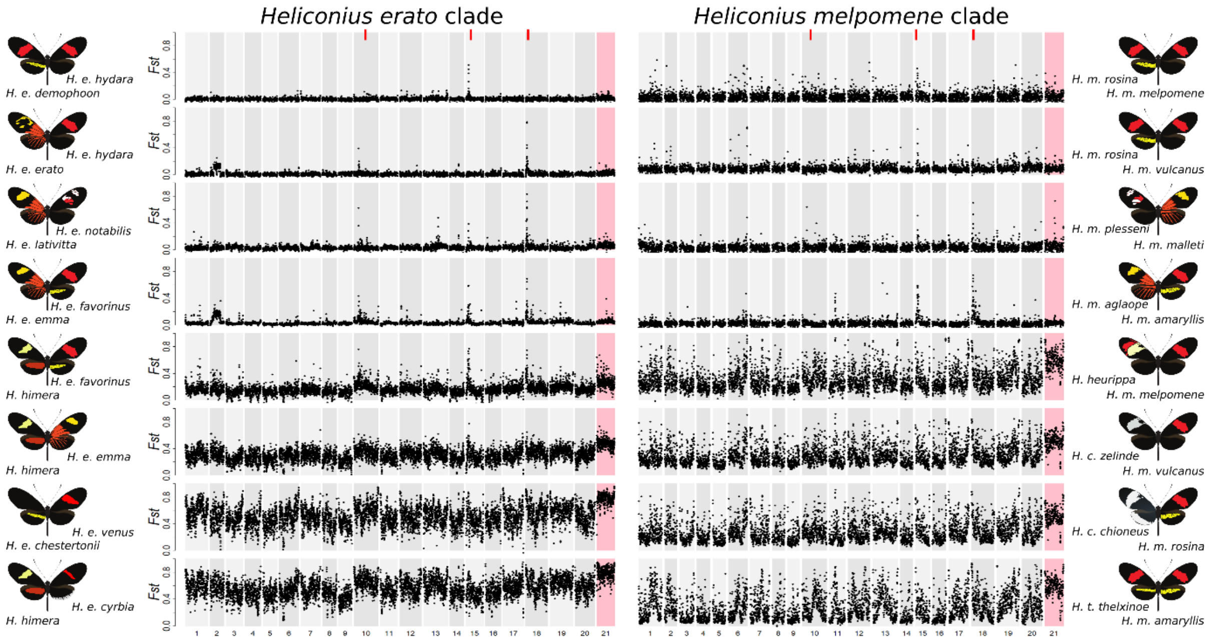

Figure S 4. F_{ST} plots of hybridizing *H. erato* and *H. melpomene* clade populations. Pairwise population comparisons include parapatric color pattern races of *H. erato* and *H. melpomene* (top four) and parapatric *H. erato* and sympatric *H. melpomene* clade populations for which there is support for assortative mating (*H. erato* - *H. himera*) as well as postmating incompatibilities (*H. erato* - *H. e. chestertonii*; *H. melpomene* – *H. cydno*; *H. melpomene* – *H. timareta*) (bottom four). Numbers 1-20 represent the autosomes. The Z chromosome is highlighted with pink shading. Red markings indicate the positions of major color pattern genes; *wntA* (chr 10), *cortex* (chr 15) and *optix* (chr 18). Values were calculated in non-overlapping 50 kb windows.

MOLECULAR ECOLOGY

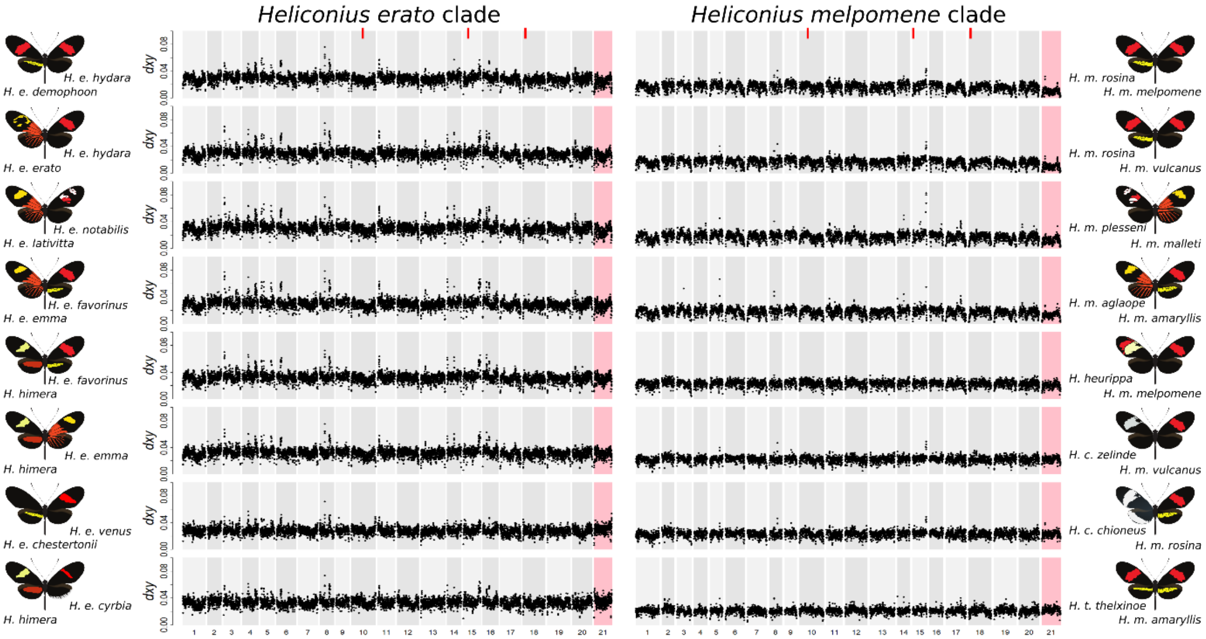

Figure S 5. d_{XY} plots of hybridizing *H. erato* and *H. melpomene* clade populations. Pairwise population comparisons include parapatric color pattern races of *H. erato* and *H. melpomene* (top four) and parapatric *H. erato* and sympatric *H. melpomene* clade populations for which there is support for assortative mating (*H. erato* - *H. himera*) as well as postmating incompatibilities (*H. erato* - *H. e. chestertonii*; *H. melpomene* – *H. cydno*; *H. melpomene* – *H. timareta*) (bottom four). Numbers 1-20 represent the autosomes. Red markings indicate the positions of major color pattern genes; *wntA* (chr 10), *cortex* (chr 15) and *optix* (chr 18). The Z chromosome is highlighted with pink shading. Values were calculated in non-overlapping 50 kb windows.

MOLECULAR ECOLOGY

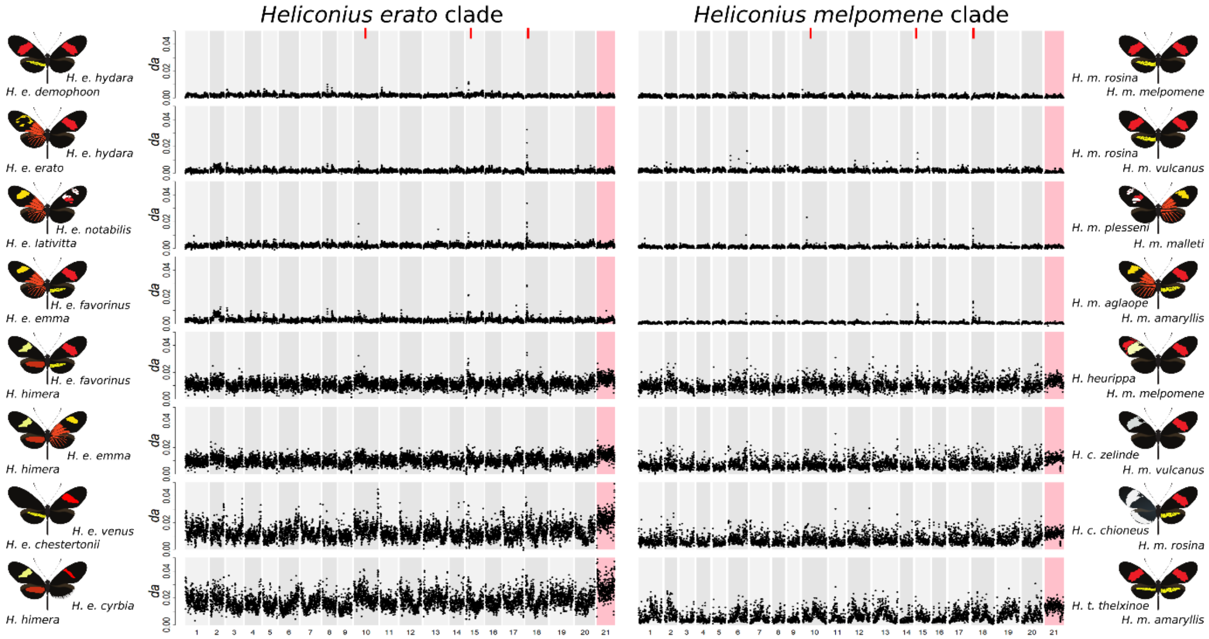

Figure S 6. d_a plots of hybridizing *H. erato* and *H. melpomene* clade populations. Pairwise population comparisons include parapatric color pattern races of *H. erato* and *H. melpomene* (top four) and parapatric *H. erato* and sympatric *H. melpomene* clade populations for which there is support for assortative mating (*H. erato* - *H. himera*) as well as postmating incompatibilities (*H. erato* - *H. e. chestertonii*; *H. melpomene* – *H. cydno*; *H. melpomene* – *H. timareta*) (bottom four). Numbers 1-20 represent the autosomes. Red markings indicate the positions of major color pattern genes; *wntA* (chr 10), *cortex* (chr 15) and *optix* (chr 18). The Z chromosome is highlighted with pink shading. Values were calculated in non-overlapping 50 kb windows.
